# Causes of death in patients with atrial fibrillation in the UK: a nationwide electronic health record study

**DOI:** 10.1093/ehjopen/oeae103

**Published:** 2024-12-13

**Authors:** Yongtong Lai, Hiroyuki Yoshimura, Nadine Zakkak, Eloi Marijon, Anwar Chahal, Gregory Y H Lip, Floriaan Schmidt, Rui Providencia

**Affiliations:** Institute of Health Informatics Research, University College London, 222 Euston Road, London NW1 2DA, UK; Institute of Health Informatics Research, University College London, 222 Euston Road, London NW1 2DA, UK; Institute of Epidemiology and Health Care, University College London, 1-19 Torrington Pl, London WC1E 7HB, UK; Cancer Intelligence, Cancer Research UK, 2 Redman Place, London E20 1JQ, UK; Division of Cardiology, European Georges Pompidou Hospital, 20 Rue Leblanc, 75015 Paris, France; Department of Cardiovascular Medicine, Mayo Clinic, 200 First St. SW, Rochester, MN 55905, USA; Center for Inherited Cardiovascular Diseases, WellSpan Health, 30 Monument Rd, York, PA 17403, USA; Barts Heart Centre, St Bartholomew's Hospital, Barts Health NHS Trust, West Smithfield, London EC1A 7BE, UK; Liverpool Centre for Cardiovascular Science at University of Liverpool, Liverpool John Moores University and Liverpool Heart and Chest Hospital, Thomas Drive, Liverpool L14 3PE, UK; Danish Center for Health Services Research, Department of Clinical Medicine, Aalborg University, Aalborg University Hospital, Selma Lagerløfs Vej 249, 9260 Gistrup, Denmark; Institute of Cardiovascular Science, University College London, 62 Huntley St, London WC1E 6DD, UK; Department of Cardiology, Amsterdam Cardiovascular Sciences, Amsterdam University Medical Center, University of Amsterdam, Meibergdreef 9, 1105 AZ Amsterdam, The Netherlands; Institute of Health Informatics Research, University College London, 222 Euston Road, London NW1 2DA, UK; Barts Heart Centre, St Bartholomew's Hospital, Barts Health NHS Trust, West Smithfield, London EC1A 7BE, UK

**Keywords:** Mortality, Arrhythmia, Sudden Cardiac Death, Cardiovascular, Women, EHR-WAS

## Abstract

**Aims:**

Causes of death remain largely unexplored in the atrial fibrillation (AF) population. We aimed to (i) thoroughly assess causes of death in patients with AF, especially those associated with sudden cardiac death (SCD) and (ii) evaluate the potential association between AF and SCD.

**Methods and results:**

Linked primary and secondary care United Kingdom Clinical Practice Research Datalink dataset comprising 6 529 382 individuals aged ≥18. We identified 214 222 patients with newly diagnosed AF, and an equivalent number of non-AF patients matched for age, sex and primary care practice. The underlying primary cause of death for each patient was assessed in the form of International Classification of Diseases Tenth Revision (ICD-10) codes and also as part of broader disease categories (i.e. ICD-10 chapters).

**Findings:**

Over a median follow-up of 2.7 (interquartile range: 0.7–6.0) years, 124 781 (58.25%) patients with AF died. Sudden cardiac death occurred in 13 923 patients with AF [6.50% patients with AF vs. 2.01% non-AF patients; odds ratio (OR) = 3.38, 95% confidence interval (CI): 3.27–3.50, *P* < 0.0001], contributing to 11.05% of all AF mortality. Diseases of the circulatory system, neoplasms and respiratory diseases explained 45% of AF mortality. Sudden cardiac death occurred more frequently in males (OR = 1.87, 95% CI: 1.80–1.93, *P* < 0.0001), and females with AF died more often of diseases of the circulatory, respiratory, digestive, and genitourinary system and less often of neoplastic disorders.

**Interpretation:**

Conditions of the circulatory system are the main driver of mortality in the AF population. Females with AF experience higher cardiovascular and respiratory mortality but die less frequently of neoplasms. The risk of SCD is higher in the AF population, occurring more frequently in males.

**Key Learning Points**

- What is already known:
  - Patients with atrial fibrillation (AF) have higher mortality and risk of sudden cardiac death (SCD) than the general population.
  - Females with AF have higher mortality, but a detailed assessment on causes of death contributing for that risk excess is currently lacking.
  - No nationwide data on AF mortality and SCD are available for the UK or other European countries.
- What this study adds:
  - We identified 2432 different primary causes of death among 259 391 study participants who died and listed the top 100 primary causes of death in the AF population.
  - We demonstrated that diseases of the circulatory system, neoplasms, and respiratory diseases explained 45% of AF mortality.
  - Sudden cardiac death occurs more frequently in males with AF, and females with AF die more often of diseases of the circulatory, respiratory, digestive and genitourinary system and less often of neoplastic disorders.

## Background

Atrial fibrillation (AF) is recognized as the most prevalent cardiac arrhythmia globally and based on data from the Global Burden of Disease it may affect over 50 million worldwide.^1^ Atrial fibrillation is associated with many cardiovascular and non-cardiovascular comorbidities,^2^ and new-onset AF has been associated with an increased mortality and morbidity from stroke, heart failure, dementia, and hospitalizations.^3^

A nationwide Korean study showed that compared with the general population, patients with AF have higher mortality risk from cardiovascular and non-cardiovascular causes.^4^ Stroke is one of the known and most feared AF-related complications^5^ but is responsible for only 7% of mortality.^3,6^ Importantly, heart failure, infections, and cancer,^6^ as well as ischaemic heart disease^3^ are thought to be the main contributors to AF mortality. Nevertheless, a detailed assessment of the most frequent primary causes of death in the AF population utilizing an electronic health records-wide association study (EHR-WAS) is currently lacking.

Sudden cardiac death (SCD) is defined as an unexpected fatal event occurring in a short time period (usually within an hour of symptom onset) in individuals with known or unknown cardiac disease.^7^ In Western countries, SCD contributes to 15–20% of all fatalities, with ischaemic heart disease, cardiomyopathies/heart failure, inherited arrhythmias, and valvular heart disease as the main contributing disease substrates.^8^ The Department of Health estimates that annually 100 000 people in the UK die suddenly due to cardiac causes, with ischaemic heart disease involved in most cases.^9,10^

A meta-analysis of observational studies and randomized controlled trials from Odutayo *et al*.^2^ showed that individuals with AF have a 46% higher risk of all-cause mortality, and 88% higher risk of SCD. However, no nationwide data are available for the UK or other European countries, and a better understanding of the substrate for the increased risk of SCD in patients with AF is required.

Among patients with AF, females have higher mortality and AF-related complications, such as stroke,^3,4,11^ although recent data suggest this sex difference AF-related stroke is less apparent.^12^ A lower risk of SCD has been reported for females across all age groups in a nationwide Danish study.^13^ Whether or not this occurs in the AF population, and what are the potential drivers for this difference, remains to be explained.

We aimed to (i) thoroughly assess the causes of death in patients with AF utilizing an EHR-WAS approach; (ii) evaluate the potential associations between AF and SCD, and (iii) investigate potential differences in causes of death between males and females with AF, utilizing a nationwide UK dataset.

## Methods

We investigated the causes of death in patients with AF compared to their age, gender, and primary care-practice matched controls without AF, as well as the association of AF with SCD, in a UK nationwide electronic health record dataset. To achieve the goal, we identified people with newly diagnosed AF from 1 January 1998 to 31 May 2016, and subsequently, matched them with patients not afflicted with AF.

### Data sources, study population, and design

Our linked data included data from primary care (Clinical Practice Research Datalink, CPRD), secondary care (Hospital Episodes Statistic, HES), and death registry data (Office for National Statistics, ONS), comprising a total of 6 529 382 participants aged 18 years or older with complete data.^3^ This dataset comprises data from ∼10% of primary care practices across the UK, including information from England, Scotland, Wales, and Northern Ireland. Data on the distribution in terms of age, gender, socioeconomic diversity, geographic location, prevalence of various health conditions, and their management accurately mirrors the overall population of the UK, and validation on accuracy, reliability, and completeness are available.^14–16^

For each incident AF patient, a matched non-AF control was selected within the same primary care practice, according to the sex and age at the index date, utilizing the exact matching method from NHS-R Community.^17^ The index date was defined as the date of initial AF diagnosis for individuals with AF, and as the earliest of the following for controls: the date of the participant's registration or the start date of data collection by the primary care-practice. The end of the study was established by the earliest date among the following circumstances: the patient died, or the date of study exit (31 May 2016).

Study variables, including baseline comorbidities, were derived based on the phenotyping algorithms of HDR-UK Phenotype platform.^18,19^ These included sociodemographic factors, comorbidities, lifestyle measurements, and prescribed medications and were selected based on expert opinion, and availability within CPRD dataset. Atrial fibrillation was diagnosed and utilized the I48 International Classification of Diseases Tenth Revision (ICD-10) code from HES, and Read codes G573200, G573400, G573500, 3272.00, G573000, G573300, G573.00, G573z00, 3273.00, 793M100, 793M300, and G573100 from CPRD.^20^ Sudden cardiac death diagnoses included ICD-10 codes I461 and I469 from HES and ONS as well as Read code G575100, as part of a previously validated SCD phenotype.^21^

Death occurrences, precise death dates, and primary causes of death were extracted from the records of the ONS. With regards to cause of death, certificates in the UK provide information on the primary cause of death (designated as 1a; ‘the disease of condition immediately causing the death’), the underlying conditions that led to the primary cause (listed as 1b, 1c, and, eventually, 1d, as a sequence of events), and contributory factors (listed as 2, and defined as other significant conditions that contributed to the death but were not part of the direct causal sequence). For this study, the immediate cause of death (i.e. 1a) for each patient was assessed in the form of ICD-10 codes and also as part of broader disease categories (i.e. ICD-10 chapters).

### Statistical analyses

Baseline characteristics of patients with AF and non-AF controls were summarized as number and percentage of people in each category, alongside with means and standard deviations for continuous variables. The differences between variables were evaluated via χ^2^ and t-tests.

Binary logistic regressions were implemented, and odds ratios (ORs) with 95% confidence intervals (CIs) were estimated for occurrence of specific causes of death for the AF group vs. the non-AF group. A *P*-value threshold of <0.05 was defined for assessing all statistical significances.

We performed an EHR-WAS, determining the top 100 associations with AF, utilizing the previously described approach.^22^ Due to numerous logistic regressions being carried through simultaneously when assessing causes of death, we applied the Bonferroni correction to adjust for multiple comparisons and adjusted the significance level of the *P*-value threshold to minimize chances of Type I error. The adjusted significance level was the *P*-value of 0.05 divided by the number of statistical tests executed—2.039 × 10^−5^ for primary diagnosis of cause of death (i.e. 0.05/2432, due to 2432 different primary diagnoses) or 0.0025 for ICD-10 chapters (i.e. 0.05/20, due to 20 different ICD-10 chapters).

Kaplan–Meier curves were utilized to visualize time-to-event, SCD, in AF vs. non-AF patients, and males vs. females with AF, and the respective hazard ratios were estimated (unadjusted, and with adjustment for age and sex). Iris plots were utilized to visualize OR of identified ICD-10 code associations, grouped by ICD-10 chapter colour.

All analyses were conducted through the R (version 4.3.0) software and statistical software for data science (STATA/MP 17.0 edition) within the UCL’s Data Safe Haven platform in compliance with the Information Governance training.

The study and access to dataset were approved by the Medicines and Healthcare products Regulatory Agency Independent Scientific Advisory Committee [17_205].

## Results

Out of a total of 6 529 382 patients of age ≥18 years with valid linked data, 214 222 patients with newly diagnosed AF met study entry criteria (see Supplementary material online, *Figure S1*), and an equivalent number of non-AF matched patients were identified. Baseline characteristics at the study entry of AF individuals and their matched controls are displayed in *Table 1*. The mean age at diagnosis was 75 ± 13, and 49% were females. Patients with AF were more often drinking alcohol or smokers/ex-smokers, two thirds had hypertension, and when compared with controls, had significantly more cardiovascular and non-cardiovascular comorbidities at the time of diagnosis.

**Table 1:** Population demographics and comorbidities at baseline

				
Variables	AF (*n* = 214 222)	Non-AF (*n* = 214 222)	Male (*n* = 217 490)	Female (*n* = 210 954)
Age	75.24 ± 12.98	75.08 ± 13.07	72.03 ± 13.16	78.39 ± 12.05
Females	105 477 (49.24)	105 477 (49.24)	–	210 954 (100)
Alcohol consumption	80 897 (37.76)	57 023 (26.62)	70 743 (32.53)	67 177 (31.84)
Current smokers	28 449 (13.28)	18 331 (8.56)	29 673 (13.64)	17 042 (8.11)
Ex-smokers	58 346 (27.24)	21 835 (10.19)	50 990 (23.44)	29 191 (13.84)
Non-smokers	100 809 (47.06)	76 888 (35.89)	76 198 (35.04)	101 499 (48.11)
Abdominal aortic aneurysm	5098 (2.38)	1715 (0.80)	5201 (2.39)	1612 (0.76)
Diabetes	36 419 (17.00)	18 260 (8.52)	30 590 (14.07)	24 089 (11.42)
Heart failure	46 614 (21.76)	10 232 (4.78)	27 294 (12.55)	29 552 (14.01)
Hypertension	136 221 (63.59)	72 643 (33.91)	97 414 (44.79)	111 450 (52.83)
Acute myocardial infarction	29 008 (13.54)	10 986 (5.13)	25 488 (11.72)	14 506 (6.88)
Peripheral arterial disease	16 945 (7.91)	6754 (3.15)	14 032 (6.45)	9667 (4.58)
Stroke	23 941 (11.18)	10 934 (5.10)	16 972 (7.80)	17 903 (8.49)
Dementia	8011 (3.74)	13 867 (6.47)	7894 (3.63)	13 984 (6.63)
Valvular heart disease	14 092 (6.58)	2397 (1.12)	8048 (3.70)	8441 (4.00)
Chronic kidney disease	36 071 (16.84)	8356 (3.90)	19 867 (9.13)	24 560 (11.64)
Oral anticoagulants	14 812 (6.91)	115 (0.05)	8364 (3.85)	6563 (3.11)
Died in community	121 279 (56.61)	112 154 (52.35)	109 942 (50.55)	123 491 (58.54)
Died in hospital	3502 (1.63)	6256 (2.92)	4445 (2.04)	5313 (2.52)

Note: age at the index date is age at AF diagnosis for patients with AF and the date of study entry for controls. Oral anticoagulants prescription at the time of diagnosis.

When segregated by sex, females were significantly older on average (78 ± 12 vs. 72 ± 13, *P* < 0.0001) but were less frequently smokers or ex-smokers. Females were more frequently hypertensive and more likely to have history of stroke, dementia, and chronic kidney disease at baseline.

During a median follow-up period of 2.7 (interquartile range: 0.7–6.0) years, 124 781 (58.25%) patients with AF, and 118 410 (55.27%) non-AF patients died (*P* < 0.0001). Regardless of whether patients had AF, the majority of patients died in the community: 56.61% died in the community, and 1.63% died in hospital, for AF cases; 52.35% vs. 2.92% for non-AF controls.

### AF vs. non-AF patients

**Figure 1:**
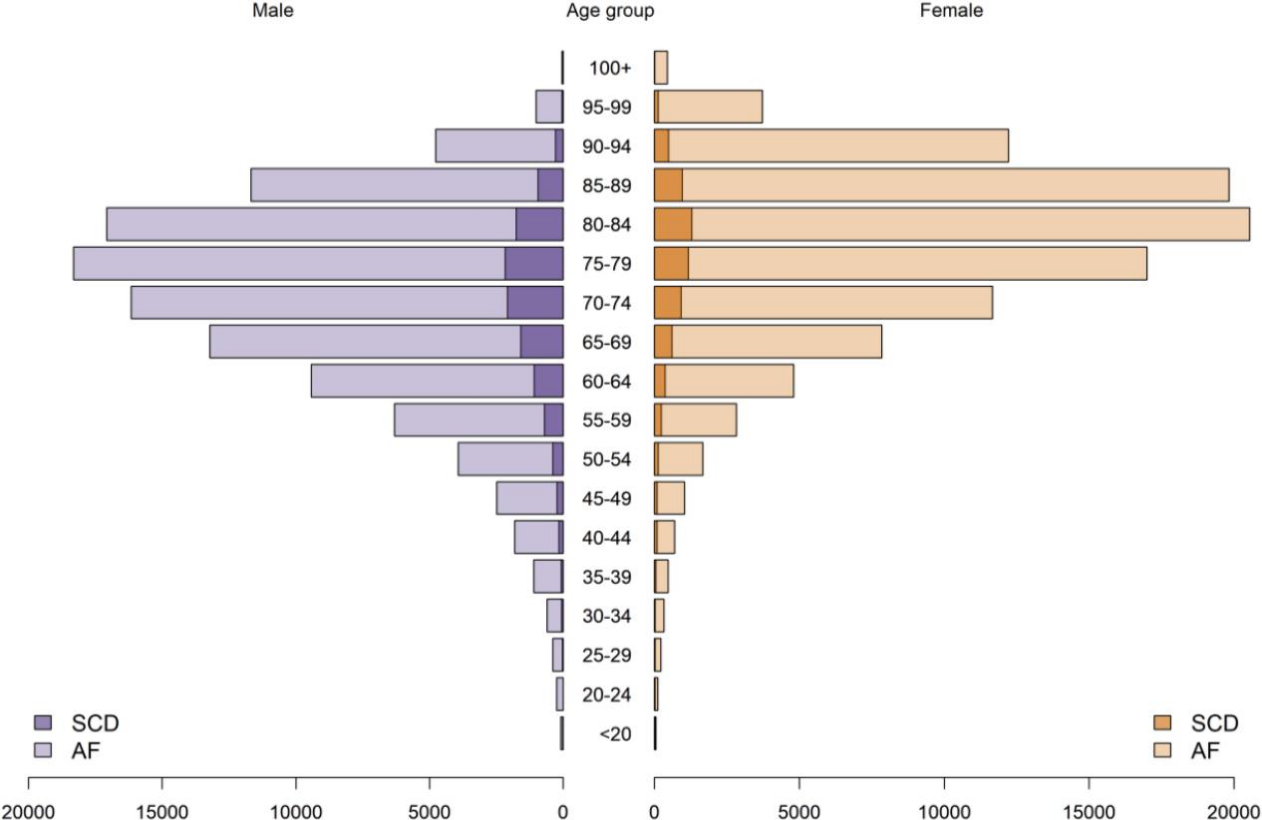
Distribution of age and gender the study population by atrial fibrillation and sudden cardiac death.

**Figure 2:**
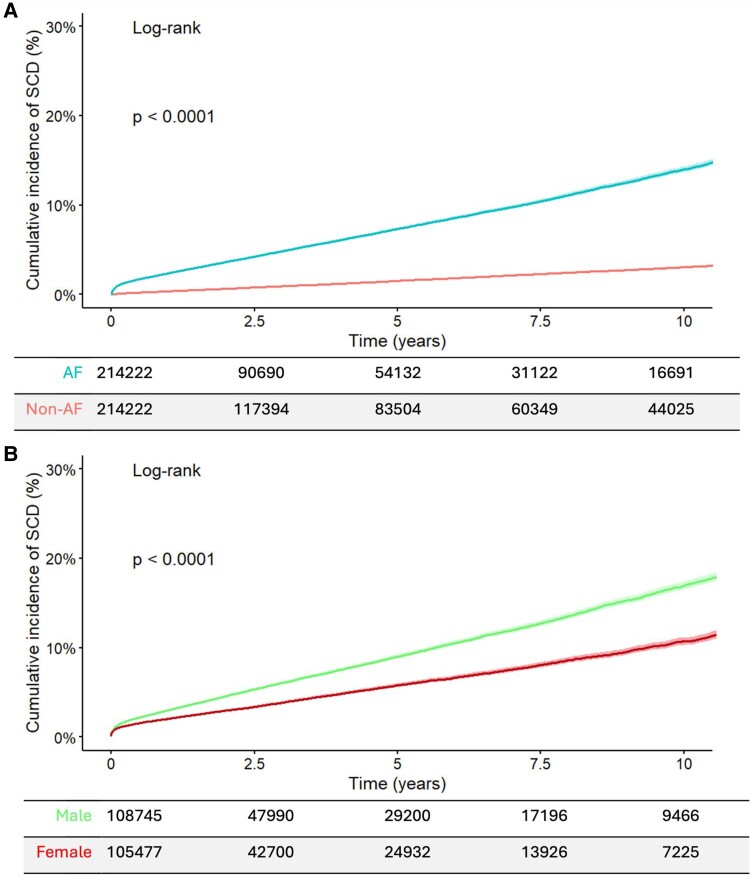
Cumulative incidence curves for sudden cardiac death in atrial fibrillation and non- atrial fibrillation groups (*A*), and atrial fibrillation males and atrial fibrillation females (*B*).

In total, 18 239 SCD events were identified among the 428 444 eligible participants. In the AF group SCD occurred in 13 923 and in non-AF patients 4316 had SCD (6.50% vs. 2.01%; OR = 3.38, 95% CI: 3.27–3.50, *P* < 0.0001). Excess in SCD mortality among patients with AF was observed both for the community (OR = 2.77, 95% CI: 2.66–2.88, *P* < 0.0001), and in hospital (OR = 1.72, 95% CI: 1.48–2.00, *P* < 0.0001). The age distribution of the AF population and SCD events is illustrated in *Figure 1*, and the cumulative incidence of SCD is represented in *Figure 2A*. Time-to-event analysis using hazard ratios confirmed an increased risk of SCD in patients with AF (unadjusted hazard ratio = 1.84, 95% CI: 1.78–1.91, and hazard ratio adjusted for age and sex = 1.85, 95% CI: 1.79–1.92). Sudden cardiac death contributed to 11.05% of all mortality cases in the AF population.

Among patients with AF, those who died from SCD were younger than those who died from other causes and more frequently males (*Table 2*). Alcohol consumption and smokers/ex-smokers were also more frequent in the SCD group. Patients with atrial fibrillation with known history of diabetes mellitus, myocardial infarction, heart failure, and valvular heart disease were more represented in the SCD group.

**Table 2:** Descriptive characteristics of the patients with atrial fibrillation (*n* = 214 222)

Variables	AF patients with SCD (*n* = 13 923)	AF patients who died (*n* = 125 993)	AF patients who survived (*n* = 88 229)	*P*-value
Age	71.99 ± 12.95	80.21 ± 9.87	68.13 ± 13.56	<0.001^a^
Females	4887 (35.10)	66 885 (53.09)	37 174 (42.13)	<0.001^a^
Alcohol consumption	5580 (40.08)	44 852 (35.60)	36 045 (40.85)	<0.001^b^
Current smokers	2598 (18.66)	16 343 (12.97)	12 106 (13.72)	<0.001^a^
Ex-smokers	4123 (29.61)	33 622 (26.69)	24 724 (28.02)	<0.001^a^
Non-smokers	5653 (40.60)	57 719 (45.81)	43 090 (48.84)	<0.001^a^
Abdominal aortic aneurysm	523 (3.76)	3715 (2.95)	1383 (1.57)	<0.001^a^
Diabetes	3070 (22.05)	23 099 (18.33)	13 320 (15.10)	<0.001^a^
Heart failure	5276 (37.89)	36 372 (28.87)	10 242 (11.61)	<0.001^a^
Hypertension	9264 (66.54)	84 100 (66.75)	52 121 (59.07)	<0.001^a^
Acute myocardial infarction	4391 (31.54)	19 912 (15.80)	9096 (10.31)	<0.001^a^
Peripheral arterial disease	1624 (11.66)	12 903 (10.24)	4042 (4.58)	<0.001^a^
Stroke	1323 (9.50)	17 559 (13.94)	6382 (7.23)	<0.001^a^
Dementia	261 (1.87)	7090 (5.63)	921 (1.04)	<0.001^a^
Valvular heart disease	1400 (10.06)	8096 (6.43)	5996 (6.80)	<0.001^a^
Chronic kidney disease	2696 (19.36)	24 034 (19.08)	12 037 (13.64)	<0.001^a^
Oral anticoagulants	1244 (8.93)	9061 (7.19)	5751 (6.52)	<0.001^a^
Died in community	9329 (96.39)	121 279 (96.26)	–	–
Died in hospital	349 (2.51)	3502 (2.78)	–	–

^a^Note: *P*-values satisfying Bonferroni correction threshold 0.05/38 = 0.0015, for the 34 comparisons of AF patients with SCD vs. AF patients who survived, and AF patients who died vs. AF patients who survived.

^b^ *P*-values satisfying Bonferroni correction threshold only for comparison of AF patients who died vs. AF patients who survived.

Diseases of the circulatory system were the predominant common cause of death, when comparing the causes of deaths by ICD chapter: 50 379 deaths in patients with AF (23.52% of all AF-deaths) and 42.221 deaths in non-AF patients. This was followed by neoplasms, accounting for 20% of deaths, and diseases of respiratory at 17%. Comparing the relative frequency of mortality explained by each ICD-10 chapter, patients with AF died more frequently due to diseases of the circulatory system (23.52% vs. 19.71% in non-AF controls; OR = 1.25, 95% CI: 1.23–1.27, *P* < 0.0001), and less frequently from neoplasms (11.06% vs. 13.71%, respectively; OR = 0.78, 95% CI: 0.77–0.80, *P* < 0.0001; see Supplementary material online, *Table S1* and Supplementary material online, *Figure S2*).

There were 2432 different primary causes of death recognized among the 259 391 people who died (adjusted significance level for multiple comparisons: 0.05/2432, *P* < 2.039 × 10^−5^). Among the top primary causes of death listed by absolute frequency in descending order, over half fell under the chapter on circulatory system diseases. The most frequent cause of death (*n* = 15 592) was ‘chronic ischaemic heart disease, unspecified’, which was twice more prevalent in the AF group (4.77% vs. 2.51%, OR = 1.94, 95% CI: 1.88–2.01, *P* < 0.0001) (*Tables 3* and *4*). This was followed by deaths due to ‘*stroke, not specified as haemorrhage or infarction’* (*n* = 13 694) and ‘*acute myocardial infarction, unspecified’* (*n* = 12 513), both (OR = 1.08, 95% CI: 1.05–1.12, *P* < 0.0001). Primary causes of death with large ORs were included in the top five, such as ‘myotonic disorders’ [0.01% (*n* = 26) vs. 0% (*n* = 1), OR = 26.00, 95% CI: 3.53–191.35], and ‘ventricular fibrillation and flutter’ [0.01% (*n* = 18) vs. 0% (*n* = 1), OR = 18.00, 95% CI: 2.41–143.66; *Table 4*]. However, due to the relatively small number of patients with deaths caused by those causes, none crossed the adjusted *P* value threshold for multiple comparisons.

**Table 3:** Top primary causes of death by descending order of absolute frequencies

Causes of death	AF *n* (%)	Non-AF *n* (%)
Chronic ischaemic heart disease, unspecified (I25.9)	10 213 (4.77)	5379 (2.51)
Stroke, not specified as haemorrhage or infarction (I64)	7115 (3.32)	6579 (3.07)
Acute myocardial infarction, unspecified (I21.9)	6499 (3.03)	6014 (2.81)
Unspecified dementia (F03)	5549 (2.59)	10126 (4.73)
Malignant neoplasm: bronchus or lung, unspecified (C34.9)	4949 (2.31)	4510 (2.11)
Bronchopneumonia, unspecified (J18.0)	4249 (1.98)	5723 (2.67)
Pneumonia, unspecified (J18.9)	4163 (1.94)	2736 (1.28)
Chronic obstructive pulmonary disease, unspecified (J44.9)	4014 (1.87)	3219 (1.5)
Chronic obstructive pulmonary disease with acute lower respiratory infection (J44.0)	2362 (1.1)	998 (0.47)
Cerebrovascular disease, unspecified (I67.9)	2324 (1.08)	3195 (1.49)
Senility (R54)	2004 (0.94)	4803 (2.24)
Congestive heart failure (I50.0)	1989 (0.93)	1048 (0.49)
Malignant neoplasm of prostate (C61)	1945 (0.91)	2739 (1.28)
Alzheimer disease, unspecified (G30.9)	1282 (0.6)	3262 (1.52)
Cerebral infarction, unspecified (I63.9)	1217 (0.57)	961 (0.45)
Malignant neoplasm: breast, unspecified (C50.9)	1209 (0.56)	1627 (0.76)
Other specified respiratory disorders (J98.8)	1169 (0.55)	1773 (0.83)
Aortic (valve) stenosis (I35.0)	1085 (0.51)	474 (0.22)
Malignant neoplasm without specification of site (C80)	1063 (0.5)	1718 (0.8)
Malignant neoplasm: colon, unspecified (C18.9)	1029 (0.48)	1284 (0.6)
Intracerebral haemorrhage, unspecified (I61.9)	981 (0.46)	728 (0.34)
Malignant neoplasm: pancreas, unspecified (C25.9)	888 (0.41)	1117 (0.52)
Peripheral vascular disease, unspecified (I73.9)	875 (0.41)	499 (0.23)
Malignant neoplasm: bladder, unspecified (C67.9)	869 (0.41)	1197 (0.56)
Other interstitial pulmonary diseases with fibrosis (J84.1)	844 (0.39)	657 (0.31)
Parkinson disease (G20)	823 (0.38)	1981 (0.92)
Unspecified acute lower respiratory infection (J22)	792 (0.37)	573 (0.27)
Sepsis, unspecified (A41.9)	653 (0.3)	364 (0.17)
Heart failure, unspecified (I50.9)	627 (0.29)	321 (0.15)
Malignant neoplasm: stomach, unspecified (C16.9)	600 (0.28)	859 (0.4)
Abdominal aortic aneurysm, ruptured (I71.3)	600 (0.28)	940 (0.44)
Chronic obstructive pulmonary disease with acute exacerbation, unspecified (J44.1)	585 (0.27)	195 (0.09)
Multiple myeloma (C90.0)	578 (0.27)	383 (0.18)
Hypertensive heart disease with (congestive) heart failure (I11.0)	564 (0.26)	266 (0.12)
Exposure to unspecified factor causing other and unspecified injury (X59.9)	491 (0.23)	668 (0.31)
Chronic ischaemic heart disease NOS (414.9)	471 (0.22)	1170 (0.55)
Myocardial degeneration (I51.5)	455 (0.21)	699 (0.33)
Acute myocardial infarction (410)	399 (0.19)	1695 (0.79)
Unspecified fall—Home (W19.0)	398 (0.19)	274 (0.13)
Bronchiectasis (J47)	371 (0.17)	186 (0.09)
Endocarditis, valve unspecified (I38)	352 (0.16)	68 (0.03)
Exposure to unspecified factor (X59.0)	347 (0.16)	239 (0.11)
Dilated cardiomyopathy (I42.0)	333 (0.16)	34 (0.02)
Malignant neoplasm, primary site unknown, so stated (C80.0)	288 (0.13)	178 (0.08)
Aortic valve disorder, unspecified (I35.9)	254 (0.12)	47 (0.02)
Cellulitis, unspecified (L03.9)	251 (0.12)	107 (0.05)
Mitral valve disease, unspecified (I05.9)	234 (0.11)	36 (0.02)
Mitral (valve) insufficiency (I34.0)	221 (0.1)	27 (0.01)
Alcoholic liver disease, unspecified (K70.9)	221 (0.1)	98 (0.05)
Essential (primary) hypertension (I10)	216 (0.1)	348 (0.16)
Unspecified diabetes mellitus—with peripheral circulatory complications (E14.5)	200 (0.09)	120 (0.06)
Malignant neoplasm, primary site unspecified (C80.9)	198 (0.09)	93 (0.04)
Mal neo bronch/lung NOS (162.9)	182 (0.08)	781 (0.36)
Cholangitis (K83.0)	173 (0.08)	101 (0.05)
Motor neuron disease (G12.2)	172 (0.08)	289 (0.13)
Calculus of gallbladder without cholecystitis (K80.2)	171 (0.08)	96 (0.04)
Chr airway obstruct NEC (496)	169 (0.08)	712 (0.33)
Ischaemic cardiomyopathy (I25.5)	164 (0.08)	30 (0.01)
Sequelae of other and unspecified cerebrovascular diseases (I69.8)	164 (0.08)	291 (0.14)
Alzheimer disease with late onset (G30.1)	159 (0.07)	395 (0.18)
Cardiomegaly (I51.7)	154 (0.07)	79 (0.04)
Cardiomyopathy, unspecified (I42.9)	137 (0.06)	18 (0.01)
Cellulitis of other parts of limb (L03.1)	134 (0.06)	57 (0.03)
Hypertensive heart and renal disease with both (congestive) heart failure and renal failure (I13.2)	122 (0.06)	50 (0.02)
Type 2 diabetes mellitus—with peripheral circulatory complications (E11.5)	121 (0.06)	55 (0.03)
Acute pancreatitis, unspecified (K85.9)	115 (0.05)	45 (0.02)
Unspecified diabetes mellitus—with renal complications (E14.2)	109 (0.05)	53 (0.02)
Other specified degenerative diseases of nervous system (G31.8)	104 (0.05)	177 (0.08)
Acute and subacute infective endocarditis (I33.0)	104 (0.05)	30 (0.01)
CHF NOS (428.0)	98 (0.05)	221 (0.1)
Other secondary pulmonary hypertension (I27.2)	94 (0.04)	9 (0)
Cholecystitis, unspecified (K81.9)	89 (0.04)	32 (0.01)
Aortic aneurysm of unspecified site, ruptured (I71.8)	88 (0.04)	161 (0.08)
Generalized and unspecified atherosclerosis (I70.9)	74 (0.03)	188 (0.09)
Alcoholic hepatic failure (K70.4)	72 (0.03)	27 (0.01)
Other ill-defined heart diseases (I51.8)	71 (0.03)	14 (0.01)
Organ-limited amyloidosis (E85.4)	67 (0.03)	22 (0.01)
Disorders of both mitral and aortic valves (I08.0)	60 (0.03)	13 (0.01)
Mitral stenosis (I05.0)	56 (0.03)	11 (0.01)
Primary pulmonary hypertension (I27.0)	51 (0.02)	8 (0)
Unspecified diabetes mellitus—with multiple complications (E14.7)	50 (0.02)	11 (0.01)
Tricuspid insufficiency (I07.1)	42 (0.02)	7 (0)
Other obesity (E66.8)	41 (0.02)	4 (0)
Cerebral atherosclerosis (I67.2)	40 (0.02)	90 (0.04)
Other forms of systemic sclerosis (M34.8)	39 (0.02)	4 (0)
Arthrosis, unspecified (M19.9)	37 (0.02)	93 (0.04)
Mal neo oesophagus NOS (150.9)	34 (0.02)	160 (0.07)
Cerebral artery occlusion, unspecified (434.9)	32 (0.01)	118 (0.06)
Diabetes mellitus without mention of complication (250.0)	31 (0.01)	149 (0.07)
Peripheral vascular disesase NOS (443.9)	31 (0.01)	117 (0.05)
Degenerative disease of nervous system, unspecified (G31.9)	29 (0.01)	81 (0.04)
Urinary tract infection NOS (599.0)	27 (0.01)	131 (0.06)
Malaise and fatigue (R53)	26 (0.01)	83 (0.04)
Traumatic amputation of arm and hand (complete) (partial) (887)	24 (0.01)	80 (0.04)
Left heart failure (428.1)	20 (0.01)	72 (0.03)
Other lymphomas (202.8)	19 (0.01)	89 (0.04)
Pneumococcal pneumonia (481)	18 (0.01)	70 (0.03)
Unspecified (402.9)	17 (0.01)	67 (0.03)
Osteoporosis (733.0)	12 (0.01)	56 (0.03)
Emphysema (492)	11 (0.01)	52 (0.02)

**Table 4:** Top primary causes of death, as measured by OR magnitude in descending order

Variables	OR (95% CI)	*P*-value
Myotonic disorders (G71.1)	25.98 (3.53, 191.35)	0.001
Ventricular fibrillation and flutter (I49.0)	18.00 (2.41, 134.66)	0.005
Disorders of both mitral and tricuspid valves (I08.1)	16.00 (2.12, 120.49)	0.007
Alcoholic hepatitis (K70.1)	14.00 (1.84, 106.32)	0.011
Chronic constrictive pericarditis (I31.1)	13.00 (1.7, 99.24)	0.013
Alcoholic cardiomyopathy (I42.6)	12.50 (2.96, 52.78)	<0.001
Codes for research or others (U99)	10.99 (2.59, 46.78)	0.001
Other secondary pulmonary hypertension (I27.2)	10.45 (5.27, 20.7)	<0.001^a^
Other obesity (E66.8)	10.25 (3.67, 28.62)	<0.001^a^
Sleep apnoea (G47.3)	10.00 (1.28, 78.01)	0.028
Dilated cardiomyopathy (I42.0)	9.81 (6.89, 13.96)	<0.001^a^
Other forms of systemic sclerosis (M34.8)	9.75 (3.48, 27.29)	<0.001^a^
Other hypertrophic cardiomyopathy (I42.2)	9.33 (2.84, 30.69)	<0.001
Mitral (valve) insufficiency (I34.0)	8.19 (5.49, 12.22)	<0.001^a^
Autoimmune hepatitis (K75.4)	8.00 (1, 63.97)	0.049
Disease of biliary tract, unspecified (K83.9)	8.00 (1, 63.97)	0.049
Ventricular tachycardia (I47.2)	8.00 (1, 63.97)	0.049
Cardiomyopathy, unspecified (I42.9)	7.62 (4.66, 12.45)	<0.001^a^
Other restrictive cardiomyopathy (I42.5)	7.50 (1.72, 32.8)	0.007
Osteomyelofibrosis (D47.4)	7.33 (2.2, 24.49)	0.001
Mitral valve disease, unspecified (I05.9)	6.51 (4.58, 9.24)	<0.001^a^
Primary pulmonary hypertension (I27.0)	6.38 (3.03, 13.43)	<0.001^a^
Tricuspid insufficiency (I07.1)	6.00 (2.7, 13.36)	<0.001^a^
Sepsis due to unspecified staphylococcus (A41.2)	6.00 (1.77, 20.36)	0.004
Aneurysm and dissection of unspecified site (I72.9)	5.99 (1.34, 26.81)	0.019
Surgical operation with anastomosis, bypass or graft (Y83.2)	5.99 (1.34, 26.81)	0.019
Other and unspecified kyphosis (M40.2)	5.50 (1.22, 24.82)	0.027
Ischaemic cardiomyopathy (I25.5)	5.47 (3.71, 8.07)	<0.001^a^
Aortic valve disorder, unspecified (I35.9)	5.41 (3.96, 7.38)	<0.001^a^
Mitral (valve) prolapse (I34.1)	5.4 (2.08, 14.02)	<0.001
Rheumatic heart disease, unspecified (I09.9)	5.33 (1.55, 18.3)	0.008
Endocarditis, valve unspecified (I38)	5.18 (4, 6.72)	<0.001^a^
Mitral stenosis (I05.0)	5.09 (2.67, 9.72)	<0.001^a^
Other ill-defined heart diseases (I51.8)	5.07 (2.86, 9)	<0.001^a^
Sarcoidosis, unspecified (D86.9)	5.00 (1.91, 13.06)	0.001
Pericardial effusion (noninflammatory) (I31.3)	5.00 (1.1, 22.82)	0.038
Aneurysm of heart (I25.3)	5.00 (1.1, 22.82)	0.038
Acute subendocardial myocardial infarction (I21.4)	5.00 (1.1, 22.82)	0.038
Other medical procedures (Y84.8)	4.67 (1.34, 16.23)	0.015
Disorders of both mitral and aortic valves (I08.0)	4.62 (2.53, 8.41)	<0.001^a^
Type 2 diabetes mellitus—with multiple complications (E11.7)	4.56 (2.21, 9.37)	<0.001
Unspecified diabetes mellitus—with multiple complications (E14.7)	4.55 (2.37, 8.73)	<0.001^a^
Obstructive hypertrophic cardiomyopathy (I42.1)	4.50 (1.52, 13.3)	0.007
Thyrotoxicosis, unspecified (E05.9)	4.13 (1.91, 8.93)	<0.001
Chronic myelomonocytic leukaemia (C93.1)	4.00 (1.5, 10.66)	0.006
Acute nephritic syndrome—unspecified (N00.9)	3.80 (1.42, 10.18)	0.008
Embolism and thrombosis of unspecified artery (I74.9)	3.75 (1.24, 11.3)	0.019
Obesity, unspecified (E66.9)	3.70 (1.84, 7.44)	<0.001
Infective myositis (M60.0)	3.67 (1.02, 13.14)	0.046
Anoxic brain damage, not elsewhere classified (G93.1)	3.60 (1.79, 7.25)	<0.001
Systemic sclerosis, unspecified (M34.9)	3.60 (1.34, 9.7)	0.011
Acute and subacute infective endocarditis (I33.0)	3.47 (2.31, 5.21)	<0.001^a^
Wegener granulomatosis (M31.3)	3.33 (1.34, 8.3)	0.01
Aortic (valve) insufficiency (I35.1)	3.31 (1.78, 6.15)	<0.001
Removal of other organ (partial) (total) (Y83.6)	3.25 (1.06, 9.97)	0.039
Fall involving bed (W06.0)	3.25 (1.06, 9.97)	0.039
Sequelae of respiratory and unspecified tuberculosis (B90.9)	3.25 (1.06, 9.97)	0.039
Diverticulum of oesophagus, acquired (K22.5)	3.17 (1.26, 7.93)	0.014
Organ-limited amyloidosis (E85.4)	3.05 (1.88, 4.93)	<0.001^a^
Chronic obstructive pulmonary disease with acute exacerbation, unspecified (J44.1)	3.01 (2.56, 3.53)	<0.001^a^
Muscular dystrophy (G71.0)	2.80 (1.01, 7.77)	0.048
Cholecystitis, unspecified (K81.9)	2.78 (1.86, 4.17)	<0.001^a^
Amyloidosis, unspecified (E85.9)	2.78 (1.3, 5.95)	0.009
Surgical operation with implant of artificial internal device (Y83.1)	2.75 (1.22, 6.18)	0.014
Amputation of limb(s) (Y83.5)	2.71 (1.14, 6.46)	0.024
Arteritis, unspecified (I77.6)	2.69 (1.51, 4.77)	<0.001
Alcoholic hepatic failure (K70.4)	2.67 (1.71, 4.15)	<0.001^a^
Cardiac arrhythmia, unspecified (I49.9)	2.67 (1.37, 5.18)	0.004
Calculus of bile duct with cholangitis (K80.3)	2.67 (1.04, 6.82)	0.04
Disease of pericardium, unspecified (I31.9)	2.67 (1.04, 6.82)	0.04
Other surgical procedures (Y83.8)	2.63 (1.16, 5.93)	0.02
Acute pancreatitis, unspecified (K85.9)	2.56 (1.81, 3.61)	<0.001^a^
Polycystic kidney, unspecified (Q61.3)	2.56 (1.18, 5.52)	0.017
Intracerebral haemorrhage, intraventricular (I61.5)	2.50 (1.28, 4.88)	0.007
Hypertensive heart and renal disease with both (congestive) heart failure and renal failure (I13.2)	2.44 (1.76, 3.39)	<0.001^a^
Chronic obstructive pulmonary disease with acute lower respiratory infection (J44.0)	2.38 (2.21, 2.57)	<0.001^a^
Pulmonary heart disease, unspecified (I27.9)	2.36 (1.17, 4.78)	0.017
Cellulitis of other parts of limb (L03.1)	2.35 (1.72, 3.21)	<0.001^a^
Cellulitis, unspecified (L03.9)	2.35 (1.87, 2.94)	<0.001^a^
Intracerebral haemorrhage in cerebellum (I61.4)	2.32 (1.35, 3.97)	0.002
Fatty (change of) liver, not elsewhere classified (K76.0)	2.31 (1.29, 4.16)	0.005
Aortic (valve) stenosis (I35.0)	2.30 (2.06, 2.56)	<0.001^a^
Alcoholic liver disease, unspecified (K70.9)	2.26 (1.78, 2.86)	<0.001^a^
Disease of intestine, unspecified (K63.9)	2.23 (1.16, 4.29)	0.016
Type 2 diabetes mellitus—with peripheral circulatory complications (E11.5)	2.20 (1.6, 3.03)	<0.001^a^
Pyothorax without fistula (J86.9)	2.19 (1.39, 3.45)	<0.001
Malignant neoplasm, primary site unspecified (C80.9)	2.13 (1.66, 2.73)	<0.001^a^
Hypertensive heart disease with (congestive) heart failure (I11.0)	2.12 (1.84, 2.46)	<0.001^a^
Peritoneal adhesions (K66.0)	2.07 (1.09, 3.92)	0.025
Unspecified diabetes mellitus—With renal complications (E14.2)	2.06 (1.48, 2.86)	<0.001^a^
Pneumonia due to *Streptococcus pneumoniae* (J13)	2.00 (1.05, 3.8)	0.034
Bronchiectasis (J47)	2.00 (1.67, 2.38)	<0.001^a^
Type 2 diabetes mellitus—with renal complications (E11.2)	1.96 (1.4, 2.74)	<0.001
Heart failure, unspecified (I50.9)	1.96 (1.71, 2.24)	<0.001^a^
Cardiomegaly (I51.7)	1.95 (1.49, 2.56)	<0.001^a^
Chronic ischaemic heart disease, unspecified (I25.9)	1.94 (1.88, 2.01)	<0.001^a^
Congestive heart failure (I50.0)	1.91 (1.77, 2.05)	<0.001^a^
Acute lymphoblastic leukaemia [ALL] (C91.0)	1.89 (1.07, 3.34)	0.029
Diffuse large B-cell lymphoma (C83.3)	1.84 (1.24, 2.73)	0.002
Acute ischaemic heart disease, unspecified (I24.9)	1.84 (1.36, 2.5)	<0.001
Unspecified diabetes mellitus—with ketoacidosis (E14.1)	1.83 (1.03, 3.26)	0.039
Surgical procedure, unspecified (Y83.9)	1.81 (1.06, 3.08)	0.029
Pneumoconiosis due to asbestos and other mineral fibres (J61)	1.81 (1.16, 2.8)	0.008
Alcoholic cirrhosis of liver (K70.3)	1.80 (1.29, 2.51)	<0.001
Sepsis, unspecified (A41.9)	1.80 (1.58, 2.04)	<0.001^a^
Calculus of gallbladder without cholecystitis (K80.2)	1.78 (1.39, 2.29)	<0.001^a^
Peripheral vascular disease, unspecified (I73.9)	1.76 (1.57, 1.96)	<0.001^a^
Sepsis due to other Gram-negative organisms (A41.5)	1.74 (1.12, 2.71)	0.014
Cerebral infarction due to embolism of cerebral arteries (I63.4)	1.74 (1.14, 2.65)	0.01
Cholangitis (K83.0)	1.71 (1.34, 2.19)	<0.001^a^
Unspecified diabetes mellitus—with peripheral circulatory complications (E14.5)	1.67 (1.33, 2.09)	<0.001^a^
Interstitial pulmonary disease, unspecified (J84.9)	1.65 (1.24, 2.18)	<0.001
Malignant neoplasm, primary site unknown, so stated (C80.0)	1.62 (1.34, 1.95)	<0.001^a^
Other forms of chronic ischaemic heart disease (I25.8)	1.61 (1.06, 2.44)	0.025
Perforation of intestine (nontraumatic) (K63.1)	1.58 (1.28, 1.96)	<0.001
Acute vascular disorders of intestine (K55.0)	1.58 (1.25, 1.98)	<0.001
Gastroenteritis and colitis of unspecified origin (A09.9)	1.57 (1.16, 2.13)	0.003
Unspecified fall—school, other institution and public administrative area (W19.2)	1.57 (1.04, 2.37)	0.033
Acute cholecystitis (K81.0)	1.56 (1.07, 2.26)	0.021
Ulcer of lower limb, not elsewhere classified (L97)	1.54 (1.21, 1.95)	<0.001
Pneumonia, unspecified (J18.9)	1.53 (1.46, 1.61)	<0.001^a^
Intracranial haemorrhage (nontraumatic), unspecified (I62.9)	1.51 (1.2, 1.9)	<0.001
Multiple myeloma (C90.0)	1.51 (1.33, 1.72)	<0.001^a^
Subdural haemorrhage (acute)(nontraumatic) (I62.0)	1.49 (1.2, 1.86)	<0.001
Chronic kidney disease (N18.0)	1.48 (1.02, 2.15)	0.041
Unspecified fall—Home (W19.0)	1.45 (1.25, 1.7)	<0.001^a^
Exposure to unspecified factor—Home (X59.0)	1.45 (1.23, 1.71)	<0.001^a^
Unspecified fall—unspecified place (W19.9)	1.42 (1.08, 1.87)	0.013
Left ventricular failure (I50.1)	1.38 (1.18, 1.62)	<0.001
Unspecified acute lower respiratory infection (J22)	1.38 (1.24, 1.54)	<0.001^a^
Other and unspecified cirrhosis of liver (K74.6)	1.38 (1.13, 1.69)	0.002
Cardiovascular disease, unspecified (I51.6)	1.36 (1.01, 1.82)	0.04
Intracerebral haemorrhage, unspecified (I61.9)	1.35 (1.23, 1.49)	<0.001^a^
Hypertensive heart disease without (congestive) heart failure (I11.9)	1.32 (1.09, 1.61)	0.005
Acute renal failure, unspecified (N17.9)	1.30 (1.04, 1.62)	0.022
Other interstitial pulmonary diseases with fibrosis (J84.1)	1.29 (1.16, 1.42)	<0.001^a^
Cerebral infarction, unspecified (I63.9)	1.27 (1.16, 1.38)	<0.001^a^
Acute myeloblastic leukaemia [AML] (C92.0)	1.26 (1.08, 1.47)	0.003
Chronic obstructive pulmonary disease, unspecified (J44.9)	1.25 (1.19, 1.31)	<0.001^a^
Rheumatoid arthritis, unspecified (M06.9)	1.24 (1.01, 1.53)	0.04
Chronic lymphocytic leukaemia of B-cell type (C91.1)	1.22 (1.01, 1.48)	0.035
Enterocolitis due to Clostridium difficile (A04.7)	1.20 (1.04, 1.38)	0.012
Malignant neoplasm of rectosigmoid junction (C19)	1.19 (1, 1.42)	0.05
Malignant neoplasm: bronchus or lung, unspecified (C34.9)	1.10 (1.06, 1.15)	<0.001^a^
Stroke, not specified as haemorrhage or infarction (I64)	1.08 (1.05, 1.12)	<0.001^a^
Acute myocardial infarction, unspecified (I21.9)	1.08 (1.05, 1.12)	<0.001^a^

^a^ *P*-values satisfied adjusted threshold of 2.039 × 10^−5^.

**Figure 3:**
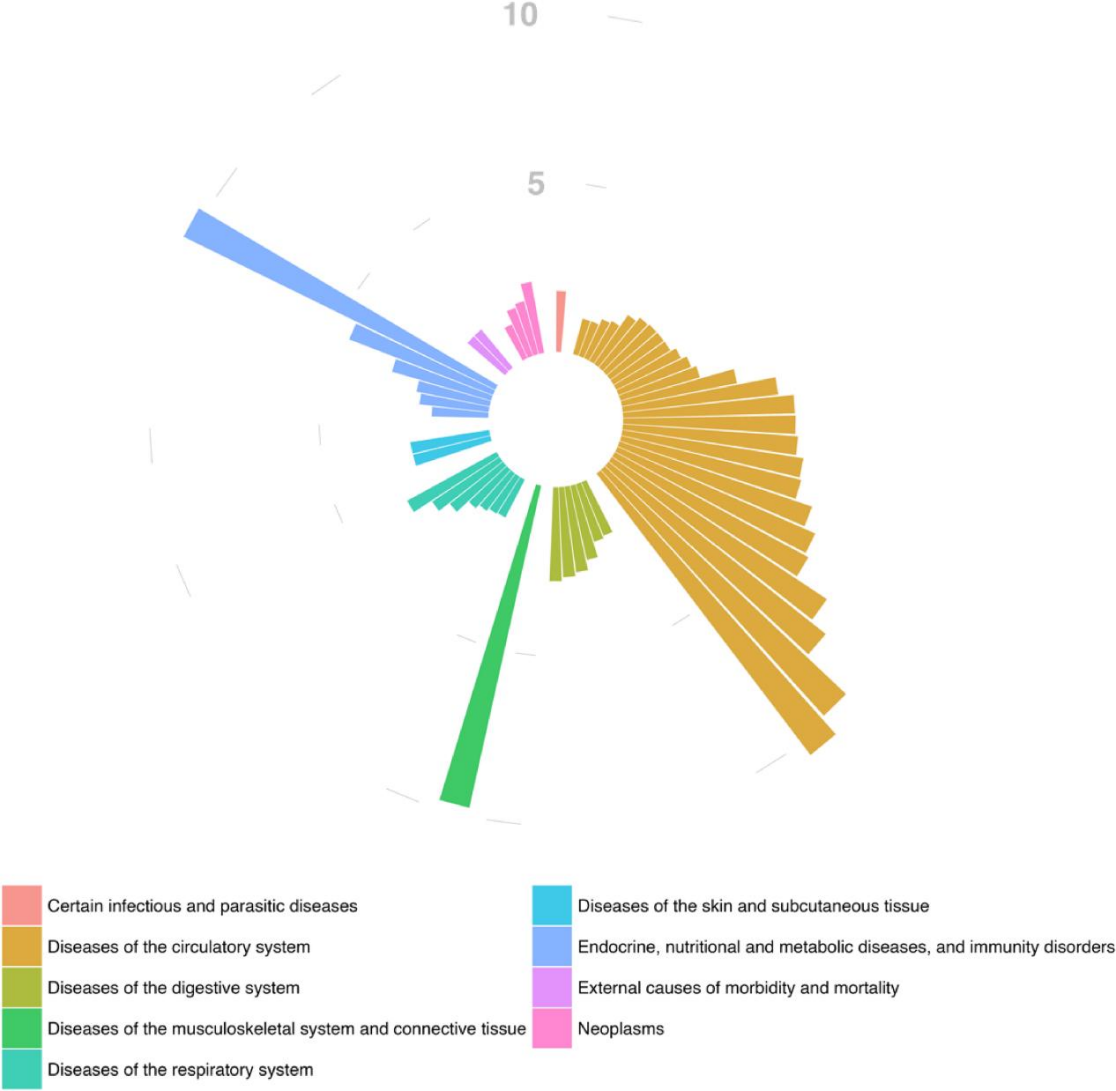
Iris plot of odds ratios for the primary causes of death that met the adjusted *P* value for multiple comparisons.

A total of 55 causes of death satisfied the criteria for the adjustment for multiple comparisons and were significantly more common in individuals with AF vs. non-AF (*Figure 3*, *Table 4*). Most of these causes of death were categorized under the diseases of circulatory system chapter (see Supplementary material online, *Figures S3* and *S4*). Cardiovascular disorders associated with SCD like ‘*dilated cardiomyopathy’*, ‘*other hypertrophic cardiomyopathy’*, ‘*cardiomyopathy unspecified’*, and ‘*ischaemic cardiomyopathy’* were more frequently causes of death in patients with AF (all with ORs >5, and meeting the adjusted *P* threshold for multiple comparisons). Besides the circulatory system conditions, other significant causes contributed to substantial odds of deaths in patients with AF, namely, ‘*other obesity’* and ‘*other forms of systemic sclerosis conditions’*, contained the second and third largest ORs meeting the adjusted *P*-value threshold.

### AF females vs. AF males

Sudden cardiac death occurred more frequently in males (OR = 1.87, 95% CI: 1.80–1.93, *P* < 0.0001; *Figure 2B*). The excess of SCD mortality in males with AF was observed across different age strata (*Figure 1*), and also for setting, with SCD in the community (OR = 1.64, 95% CI: 1.57–1.71, *P* < 0.0001), and SCD occurring in hospital (OR = 1.26, 95% CI: 1.02–1.55, *P* = 0.0339) being more frequent in males. Time-to-event analysis using hazard ratios confirmed an increased risk of SCD in males with AF (unadjusted hazard ratio = 1.68, 95% CI: 1.62–1.74, and hazard ratio adjusted for age = 1.60, 95% CI: 1.53–1.65).

Among patients with AF, females experienced higher all-cause mortality than males (OR = 1.44, 95% CI: 1.41–1.46, *P* < 0.0001). Comparisons of cause of death by organ system or disease group, as assessed by ICD-10 chapters, showed that females with AF died more often of diseases of the circulatory (OR = 1.32, 95% CI: 1.29–1.35, *P* < 0.0001), respiratory (OR = 1.15, 95% CI: 1.11–1.19, *P* < 0.0001), digestive (OR = 1.40, 95% CI: 1.33–1.49, *P* < 0.0001) and genitourinary system (OR = 1.43, 95% CI: 1.33–1.55, *P* < 0.0001), and mental and behavioural disorders (OR = 1.84, 95% CI: 1.74–1.94, *P* < 0.0001), and less often of neoplastic disorders (OR = 0.71, 95% CI: 0.69–0.74, *P* < 0.0001) when compared with males (see Supplementary material online, *Table S2*).

A detailed analysis of cause of mortality in females with AF compared with males is presented in Supplementary material online, *Table S3*, with 33 causes of death meeting the adjusted *P*-value cut-off for multiple comparisons. Females with AF died more often than males of ‘*stroke, not specified as haemorrhage or infarction’* (OR = 2.04, 95% CI: 1.90–2.16, *P* < 0.0001), ‘*unspecified dementia’* (OR = 2.02, 95% CI: 1.90–2.16, *P* < 0.0001), ‘*bronchopneumonia, unspecified’* (OR = 1.60, 95% CI: 1.49, 1.71, *P* < 0.0001)*, ‘congestive heart failure’* (OR = 1.61, 95% CI: 1.47–1.75, *P* < 0.0001), ‘*heart failure, unspecified’* (OR = 1.37, 95% CI: 1.16–1.59, *P* < 0.0001), ‘*hypertensive heart disease with congestive heart failure’* (OR = 1.37, 95% CI: 1.15–1.61, *P* < 0.0001), ‘*aortic valve stenosis’* (OR = 1.43, 95% CI: 1.27–1.62, *P* < 0.0001), ‘*mitral valve disease, unspecified’* (OR = 2.63, 95% CI: 1.98–3.49, *P* < 0.0001), ‘*endocarditis, valve unspecified’* (OR = 1.81, 95% CI: 1.45–2.24, *P* < 0.0001), ‘*bronchiectasis’* (OR = 1.72, 95% CI: 1.39–2.13, *P* < 0.0001), and ‘*cellulitis’* (OR = 1.78, 95% CI: 1.38–2.31, *P* < 0.0001), among others.

## Discussion

Utilizing nationwide UK electronic health records, our principal findings are as follows: (i) there was a 3.38-fold higher odds of SCD in patients with AF, contributing to over 10% of all deaths in this patient group; (ii) conditions of the circulatory system (ischaemic heart disease, stroke, and acute myocardial infarction being the most frequent primary diagnoses in death certificates) were the main driver of mortality in the AF population, causing over a third of all deaths, while neoplastic disorders and conditions of the respiratory system were the second and third main causes of mortality, causing a fifth and a sixth of mortality, respectively; (iii) ischaemic heart disease, stroke, and acute myocardial infarction were the most frequent primary diagnoses in death certificates. Important differences between females and males with AF were observed, with SCD being nearly twice as frequent in males, whilst females with AF tend to die more of diseases of the circulatory, respiratory, digestive, and genitourinary system, as well as more due to mental and behavioural disorders, and less often due to neoplastic disorders when compared to males.

Our findings of increased risk of cardiovascular mortality and SCD among individuals with AF, are also consistent with reports from Taiwan.^23^ Lee *et al*.^4^ using nationwide data from Korea assessed mortality in 15 411 patients with AF and differences in males and females. Similarly to our study, Lee and colleagues described a higher mortality from diseases of the circulatory system in the AF population. A subsequent analysis of the Korean cohort showed a 3-fold higher risk of SCD in patients with AF but observed no sex interaction for that outcome.^24^

Kim *et al*.^25^ utilizing the Korean National Health Insurance Service database suggested that patients with AF were more likely to experience ventricular fibrillation, ventricular tachycardia and ventricular flutter. This aligns with our findings, with ‘*ventricular fibrillation or flutter’* and ‘*ventricular tachycardia’* being observed more frequently as primary causes of death in patients with AF (OR = 18 and OR = 8, respectively).

A systematic review of 30 cohort studies had previously shown a higher-risk of all-cause and cardiovascular mortality in females with AF.^26^ Our study reproduced these findings and provided further detail on the specific causes that affect females with AF more frequently than males: congestive heart failure, stroke, vascular dementia, aortic stenosis, and endocarditis. On the other hand, we observed a lower rate of SCD in females with AF, which merits further investigation. Despite previous suggestions of hormonal protection genetic and lifestyle factors, lower incidence of ischaemic heart disease, differences in cardiac structure, function or electrical activity, or earlier and more frequent healthcare utilization,^27–30^ as protective factors for SCD in females, a definitive answer is still warranted. Interestingly, females in our sample were less frequently smokers or ex-smokers and had lower prevalence of ischaemic heart disease at baseline, which corroborates some of the abovementioned hypotheses.

Waldmann *et al*.^31^ suggested that coronary heart disease and heart failure were the two most frequent substrates for SCD in the AF population. In our study, we observed higher mortality from cardiovascular diseases in females with AF, with comparable mortality for ischaemic heart disease vs. males, and higher mortality from congestive heart failure. Nevertheless, interpretation of these observations is complex and must be seen at light of competing risk: it is possible that males may die of SCD in earlier stages of heart failure and hence some do not evolve into more advanced stages or live long enough to die of pump failure. On the other hand, females are more likely to die during a congestive heart failure admission and hence do not live long enough to experience a SCD event.

The findings of the current study reinforce the need for aggressive cardiovascular risk factor control and management to tackle mortality in the AF population. Primary prevention efforts, such as strict blood pressure and cholesterol management, as well as the encouragement of lifestyle changes like quitting smoking and drinking, may contribute to a decrease the mortality. The increased risk of SCD in the AF population prompts the need for improvement of risk stratification in this patient group and further research to understand which patients are more likely to experience SCD and whether or not any intervention may lower this risk.

Indeed, current guidelines globally have advocated a more holistic or integrated care approach to AF management,^32^ given the improved outcomes seen with adherence to such an approach, including lower mortality.^33^

### Strengths and limitations

Strengths of our investigation include a broad assessment of the top primary causes of death, and investigation of SCD with a previously validated definition in a large population representative of the UK.^20^ Our modified *P*-value threshold with adjustment for multiple comparisons reduced the chances of type I error.

However, the study limitations need to be highlighted. First, the utilization of ICD diagnosis codes for causes of death may fail to capture the complexity of events in some cases, and some variability may occur depending on the coding physician. Thus, some causes of death might be underestimated. Despite this, existing studies had proven the validation of the use of the terminology and phenotypes.^3,21,34^ Second, our study might not be able to assess all potential risk factors or diseases, but we attempted to minimize possible confounding by matching patients with AF with non-AF controls based on age, sex, and GP practice. Third, despite adjusting for age, we cannot rule out that age differences and survival bias may have played a role in some of the observed differences (e.g. was SCD observed more often in males because the women who would experience SCD died before they developed AF and/or entered the study?). Fourth, we did not perform a competing risk analysis and, as such, our findings need to be interpreted with caution. When assessing causes of death, it is accepted that if patients die from one cause, they are no longer at risk of dying from a different cause, thereby influencing the observed results for other causes of death. This might explain why for some observed associations, an OR of less than 1.0, suggesting that fewer patients with AF died of a specific cause, may in fact be a result of patients having died from a different cause instead, as reflected by a distinct ICD-10 code. Therefore, the presence of an OR less than 1.0 may not necessarily indicate a lower risk and can be a result of the competing risks not accounted for in the analysis. Fifth, for a broader scoping of the causes of mortality in patients with AF, we utilized an approach analogous to genome-wide association studies. Specifically, we conducted 2432 binary logistic regressions, one for each of the identified primary causes of death. Notably, we also performed two time-to-event analyses, and the resulting hazard ratios were consistent with the findings from the binary logistic regressions. Finally, this study does not allow to infer causality for the association between AF and SCD. It remains to be clarified if AF is driving SCD or if it reflects other comorbidities that associate with SCD (e.g. myocardial infarction and heart failure). Future studies utilizing Mendelian randomization may address this knowledge gap.

## Conclusion

Conditions of the circulatory system are the main driver of mortality in the AF population and cause over a third of all deaths. Females with AF experience higher cardiovascular and respiratory mortality but die less frequently of neoplasms.

The risk of SCD is higher in the AF population, with 10% of patients with AF expected to suffer from SCD. Sudden cardiac death is nearly twice as frequent in males with AF.

## Supplementary Material

oeae103_Supplementary_Data

## Data Availability

Data for this study were provided by United Kingdom’s Medicines and Healthcare products Regulatory Agency following approval by the Independent Scientific Advisory Committee [17_205], and can be made available to other researchers following application via the Clinical Practice Research Datalink (CPRD) website (https://www.cprd.com/).
