# Supplementary material for: Causes of death in patients with atrial fibrillation in the UK: a nationwide electronic health record study
Source: Eur Heart J Open. 2024 Dec 13;5(1):oeae103. doi: 10.1093/ehjopen/oeae103 (PMC11711847; doi:10.1093/ehjopen/oeae103)
Supplement: oeae103_Supplementary_Data [file oeae103_supplementary_data.docx]

**Supplementary Material**

**Figure S.1.** Flow-chart of the study population. Index date is the date of study entry for non-AF controls and the date of the first AF diagnosis for AF individuals.

Participants above the age of 18 with linked clinical data enrolled in primary care

6,529,382

AF patients with verified records

341,553

Filtering participants with a diagnosis of AF

AF patients with valid entry and exit dates

214,222

Non-AF patients with valid entry and exit dates

214,222

Matching age, gender, and GP-Practice at the index date (1:1 ratio)

With SCD

13,923

Without SCD

200,299

With SCD

4,316

Without SCD

209,906

**Figure S.2.** Causes of death by ICD-10 Chapter: AF patients (Panel A) and non-AF controls (Panel B)

**A.**


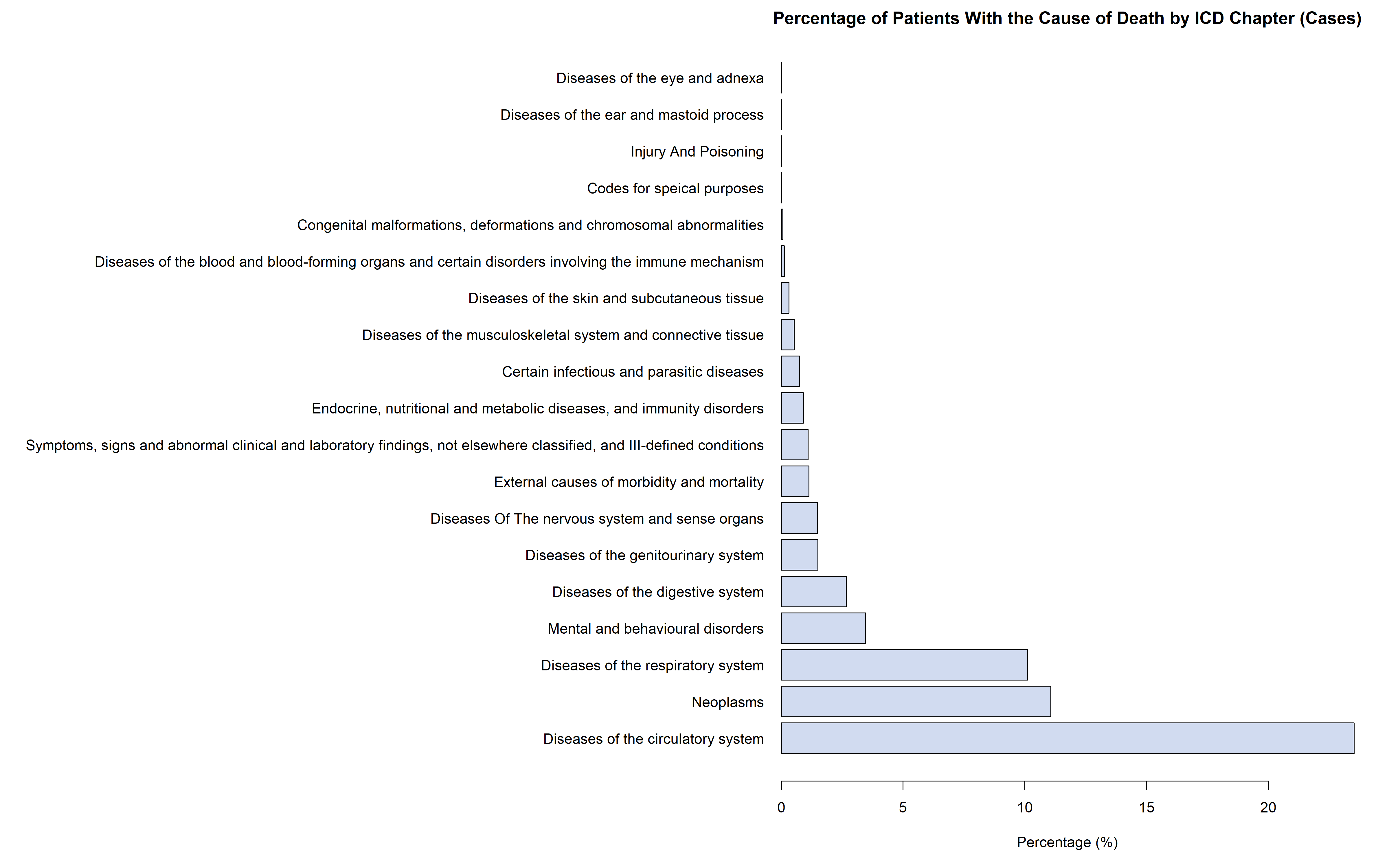


**B.**


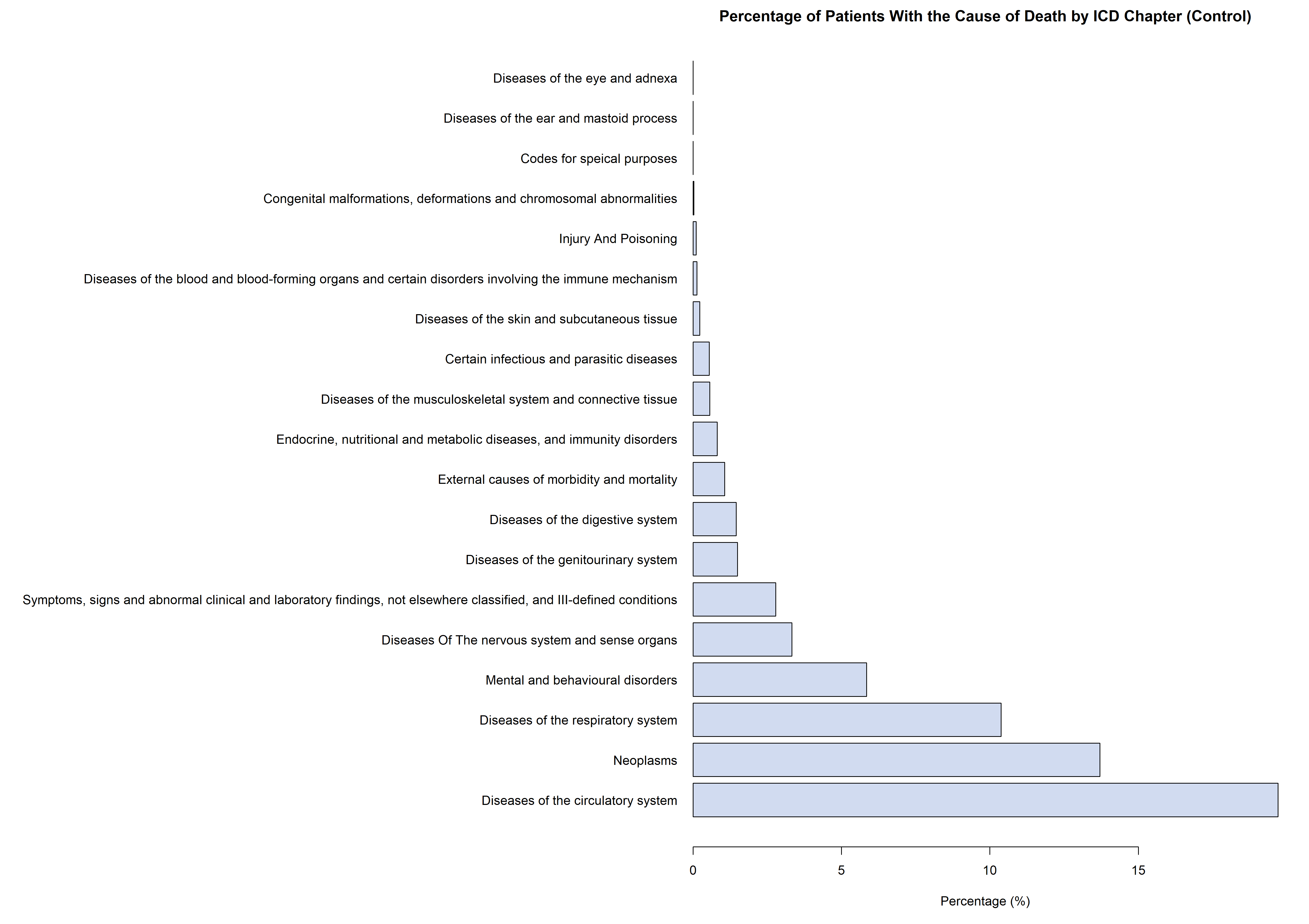


**Figure S.3.** Iris plot of odds ratios for the most frequent primary causes of death at unadjusted significance level of 0.05.


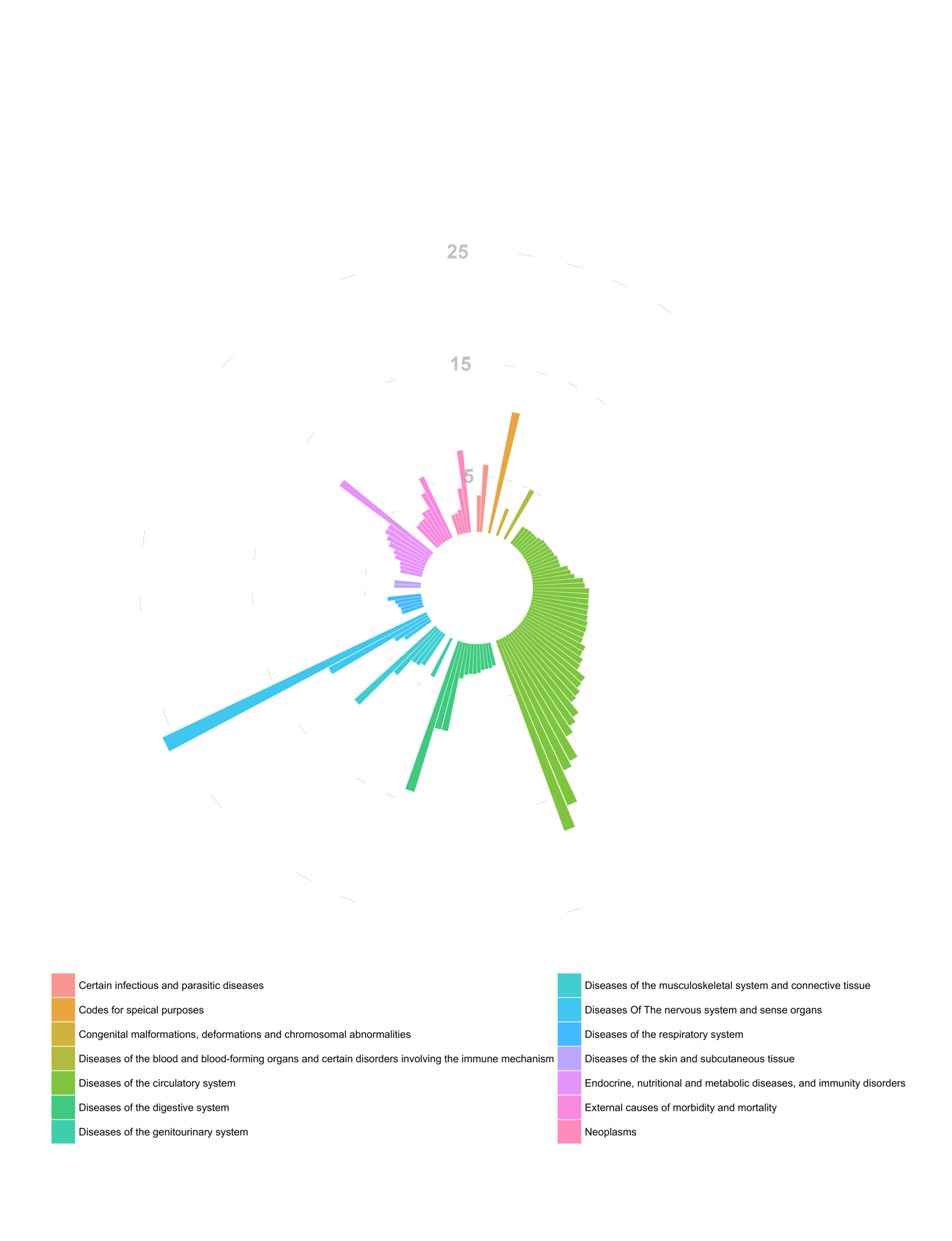


**Figure S.4.** Top 30 primary causes of death in AF patients (Panel A) and non-AF controls (Panel B) meeting the adjusted significance threshold for multiple comparisons

**A.**


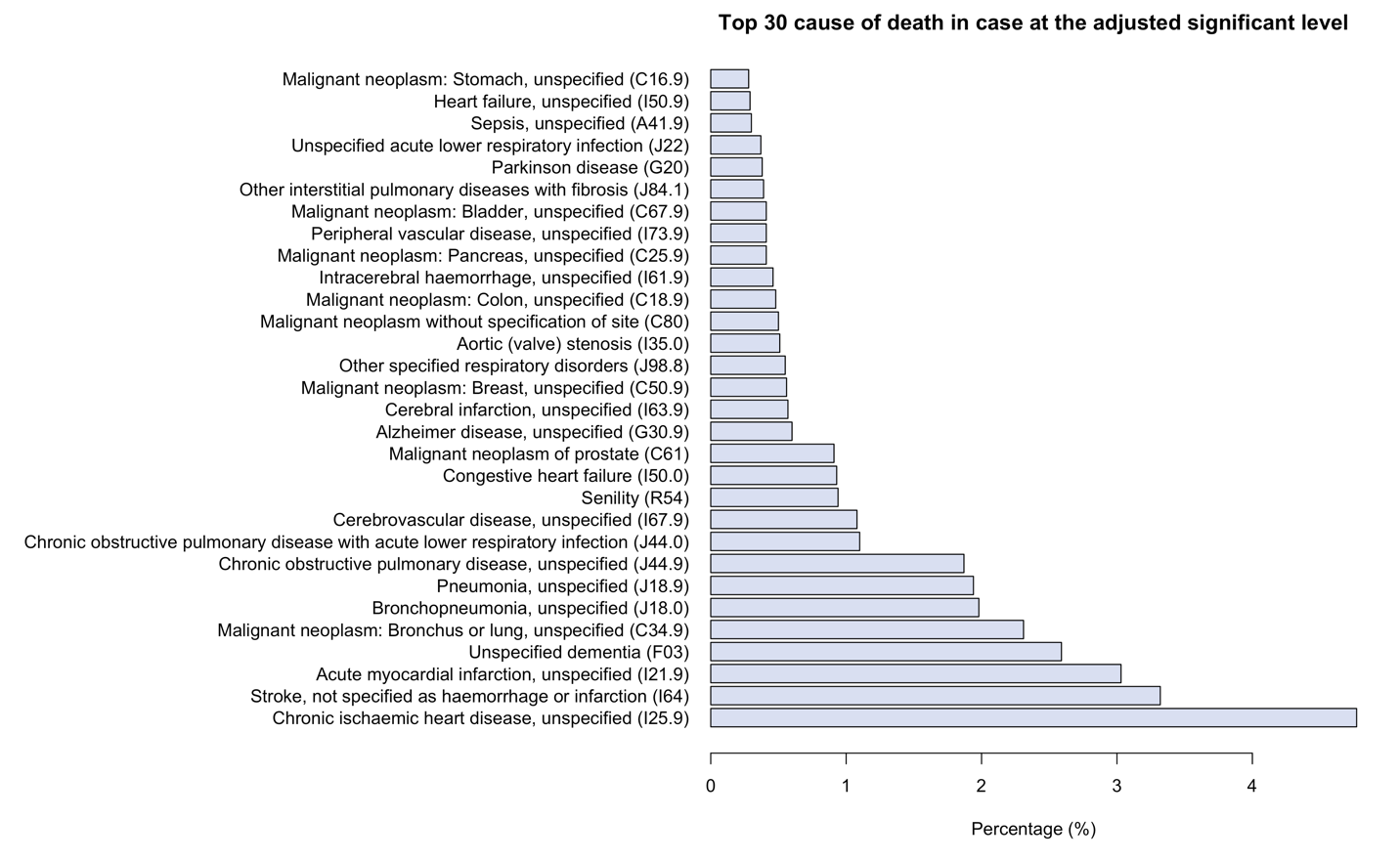


**B.**


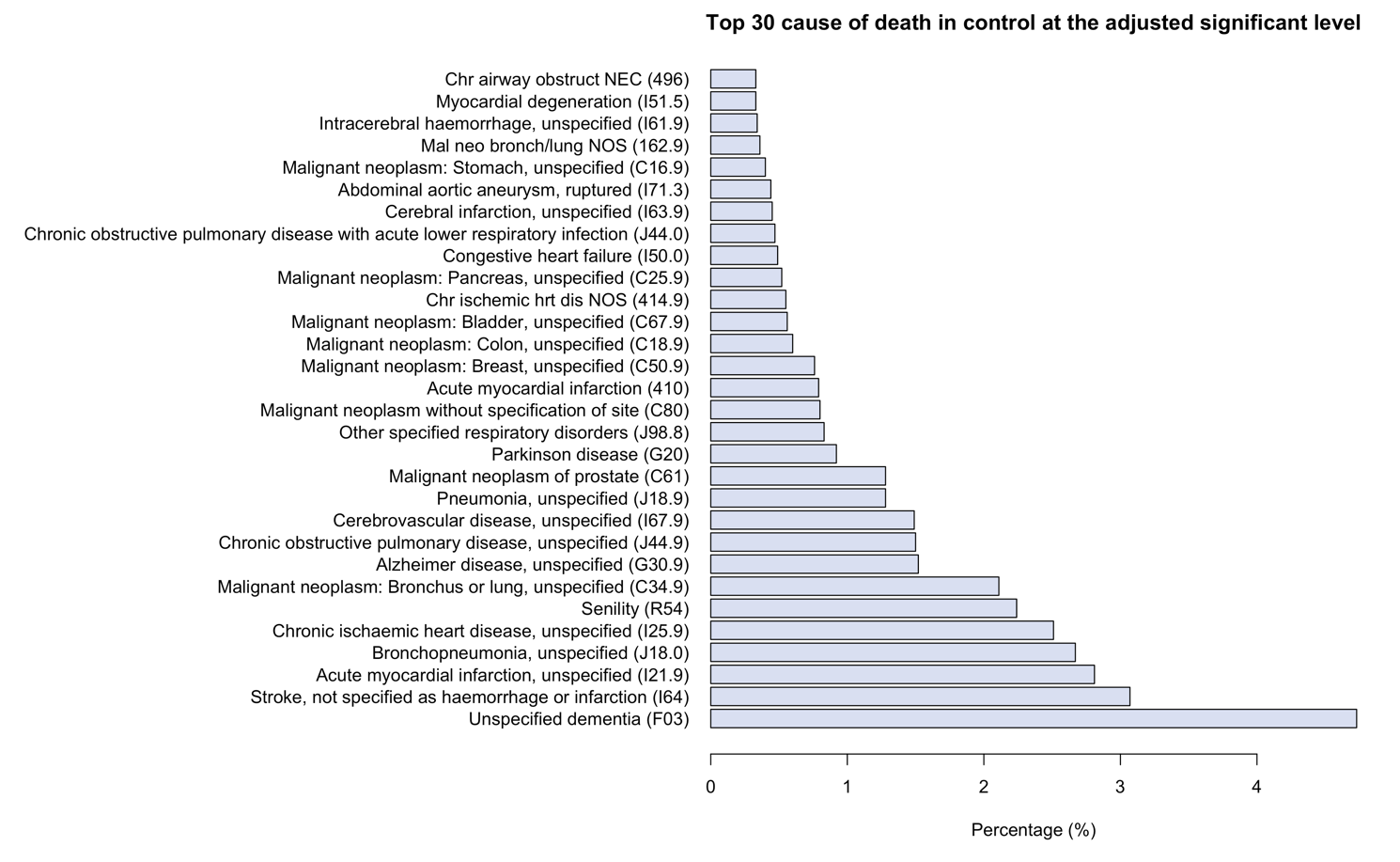


**Table S.1.** Frequency of Cause of death by ICD chapter (AF versus non-AF)

| **Causes of Death** | **AF**  **N(%)** | **Non-AF**  **N(%)** | **OR (95%CI)** | **P-value** |
| --- | --- | --- | --- | --- |
| I - Diseases of the circulatory system | 50379 (23.52) | 42221 (19.71) | 1.25  (1.23, 1.27) | <0.001* |
| C - Neoplasms | 23687 (11.06) | 29368 (13.71) | 0.78  (0.77, 0.8) | <0.001* |
| J - Diseases of the respiratory system | 21684 (10.12) | 22246 (10.38) | 0.97  (0.95, 0.99) | 0.005 |
| F - Mental and behavioural disorders | 7406 (3.46) | 12526 (5.85) | 0.58  (0.56, 0.59) | <0.001* |
| K - Diseases of the digestive system | 5730 (2.67) | 3120 (1.46) | 1.86  (1.78, 1.94) | <0.001* |
| N - Diseases of the genitourinary system | 3218 (1.5) | 3221 (1.5) | 1.00  (0.95, 1.05) | 0.97 |
| G - Diseases of the nervous system and sense organs | 3190 (1.49) | 7140 (3.33) | 0.44  (0.42, 0.46) | <0.001* |
| V-Y - External causes of morbidity and mortality | 2428 (1.13) | 2299 (1.07) | 1.06  (1.00, 1.12) | 0.059 |
| R - Symptoms, signs and abnormal clinical and laboratory findings, not elsewhere classified, and III-defined conditions | 2354 (1.1) | 5977 (2.79) | 0.39  (0.37, 0.41) | <0.001* |
| E - Endocrine, nutritional and metabolic diseases, and immunity disorders | 1952 (0.91) | 1751 (0.82) | 1.12  (1.05, 1.19) | <0.001* |
| A - Certain infectious and parasitic diseases | 1616 (0.75) | 1173 (0.55) | 1.38  (1.28, 1.49) | <0.001* |
| M - Diseases of the musculoskeletal system and connective tissue | 1143 (0.53) | 1224 (0.57) | 0.93  (0.86, 1.01) | 0.095 |
| L - Diseases of the skin and subcutaneous tissue | 689 (0.32) | 499 (0.23) | 1.38  (1.23, 1.55) | <0.001* |
| D50-89 - Diseases of the blood and blood-forming organs and certain disorders involving the immune mechanism | 265 (0.12) | 288 (0.13) | 0.92  (0.78, 1.09) | 0.328 |
| Q - Congenital malformations, deformations and chromosomal abnormalities | 156 (0.07) | 62 (0.03) | 2.52  (1.88, 3.38) | <0.001* |
| S-T - Injury And Poisoning | 40 (0.02) | 227 (0.11) | 0.18  (0.13, 0.25) | <0.001* |
| U - Codes for special purposes | 37 (0.02) | 9 (0) | 4.11  (1.98, 8.52) | <0.001* |
| H00-59 - Diseases of the eye and adnexa | 5 (0) | 4 (0) | 1.25  (0.34, 4.65) | 0.739 |
| H60-95 - Diseases of the ear and mastoid process | 4 (0) | 1 (0) | 4  (0.45, 35.79) | 0.215 |

*Note: P-values satisfying Bonferroni correction threshold 0.05/20 = 0.0025

**Table S.2.** Frequency of Cause of death by ICD chapter (AF females versus AF males)

| **Causes of Death** | **AF females**  **N(%)** | **AF males**  **N(%)** | **OR (95%CI)** | **P-value** |
| --- | --- | --- | --- | --- |
| I - Diseases of the circulatory system | 27481 (27.16) | 22897 (22.03) | 1.32  (1.29, 1.35) | <0.001* |
| C – Neoplasms | 9940 (9.7) | 13747 (13.07) | 0.71  (0.69, 0.74) | <0.001* |
| J - Diseases of the respiratory system | 11374 (11.11) | 10310 (9.81) | 1.15  (1.11, 1.19) | <0.001* |
| F - Mental and behavioural disorders | 4717 (4.22) | 2689 (2.34) | 1.84  (1.74, 1.94) | <0.001* |
| K - Diseases of the digestive system | 3297 (3.16) | 2433 (2.27) | 1.40  (1.33, 1.49) | <0.001* |
| R - Symptoms, signs and abnormal clinical and laboratory findings, not elsewhere classified, and III-defined conditions | 1832 (1.81) | 5222 (0.5) | 3.65  (3.28, 4.05) | <0.001* |
| N - Diseases of the genitourinary system | 1871 (1.8) | 1347 (1.26) | 1.43  (1.33, 1.55) | <0.001* |
| G - Diseases of the nervous system and sense organs | 1615 (1.46) | 1575 (1.39) | 1.05  (0.97, 1.14) | 0.1960 |
| V-Y - External causes of morbidity and mortality | 1312 (1.28) | 1116 (1.06) | 1.21  (1.11, 1.32) | <0.001* |
| E - Endocrine, nutritional and metabolic diseases, and immunity disorders | 1017 (0.96) | 935 (0.86) | 1.12  (1.01, 1.23) | 0.0251 |
| A - Certain infectious and parasitic diseases | 930 (0.88) | 687 (0.64) | 1.39  (1.25, 1.55) | <0.001* |
| M - Diseases of the musculoskeletal system and connective tissue | 790 (0.77) | 353 (0.34) | 2.31  (2.02, 2.65) | <0.001* |
| L - Diseases of the skin and subcutaneous tissue | 477 (0.47) | 212 (0.2) | 2.32  (1.95, 2.77) | <0.001* |
| D50-89 - Diseases of the blood and blood-forming organs and certain disorders involving the immune mechanism | 153 (0.15) | 112 (0.11) | 1.40  (1.07, 1.82) | 0.0139 |
| Q - Congenital malformations, deformations and chromosomal abnormalities | 77 (0.07) | 79 (0.07) | 0.99  (0.69, 1.42) | 0.9704 |
| S-T - Injury and Poisoning | 26 (0.03) | 14 (0.02) | 1.98  (1.04, 3.78) | 0.0380 |
| U - Codes for special purposes | 13 (0.01) | 24 (0.02) | 0.56  (0.27, 1.18) | 0.1280 |
| H00-59 - Diseases of the eye and adnexa | 3 (0) | 2 (0) | 1.55  (0.26, 9.26) | 0.6329 |
| H60-95 - Diseases of the ear and mastoid process | 1 (0) | 3 (0) | 0.34  (0.04, 3.29) | 0.3532 |

*Note: P-values satisfying Bonferroni correction threshold 0.05/20 = 0.0025

**Table S.3.** Top primary causes of death, as measured by descending order of absolute frequencies in AF females vs AF males

| **ICD-10 code** | **Cause of Death** | **AF females**  **N(%)** | **AF males**  **N(%)** | **OR (95%CI)** | **P value** |
| --- | --- | --- | --- | --- | --- |
| I25.9 | Chronic ischaemic heart disease, unspecified (I25.9) | 4984 (4.73) | 5229 (4.81) | 0.99 (0.94, 1.02) | 0.3657 |
| I64 | Stroke, not specified as haemorrhage or infarction (I64) | 4697 (4.61) | 2418 (2.31) | 2.04 (1.90, 2.16) | <0.0001* |
| F03 | Unspecified dementia (F03) | 3660 (3.26) | 1889 (1.64) | 2.02 (1.90, 2.16) | <0.0001* |
| J18.0 | Bronchopneumonia, unspecified (J18.0) | 2576 (2.54) | 1673 (1.61) | 1.60 (1.49, 1.71) | <0.0001* |
| J18.9 | Pneumonia, unspecified (J18.9) | 2409 (2.38) | 1754 (1.69) | 1.42 (1.33, 1.52) | <0.0001* |
| J44.0 | Chronic obstructive pulmonary disease with acute lower respiratory infection (J44.0) | 994 (0.94) | 1368 (1.26) | 0.74 (0.69, 0.81) | <0.0001* |
| I67.9 | Cerebrovascular disease, unspecified (I67.9) | 1430 (1.35) | 893 (0.82) | 1.65 (1.51, 1.82) | <0.0001* |
| I50.0 | Congestive heart failure (I50.0) | 1210 (1.15) | 779 (0.72) | 1.61 (1.47, 1.75) | <0.0001* |
| F01.9 | Vascular dementia, unspecified (F01.9) | 939 (0.89) | 684 (0.63) | 1.41 (1.27, 1.58) | <0.0001* |
| N39.0 | Urinary tract infection, site not specified (N39.0) | 1142 (1.08) | 660 (0.61) | 1.78 (1.61, 1.98) | <0.0001* |
| I48 | Atrial fibrillation and flutter (I48) | 1561 (1.48) | 641 (0.59) | 2.53 (2.28, 2.80) | <0.0001* |
| G30.9 | Alzheimer disease, unspecified (G30.9) | 816 (0.78) | 466 (0.46) | 1.80 (1.58, 2.06) | <0.0001* |
| I35.0 | Aortic (valve) stenosis (I35.0) | 631 (0.62) | 454 (0.44) | 1.43 (1.27, 1.62) | <0.0001* |
| J98.8 | Other specified respiratory disorders (J98.8) | 740 (0.70) | 450 (0.43) | 1.69 (1.49, 1.93) | <0.0001* |
| I63.9 | Cerebral infarction, unspecified (I63.9) | 772 (0.76) | 445 (0.43) | 1.79 (1.57, 2.03) | <0.0001* |
| R54 | Senility (R54) | 1591 (1.58) | 413 (0.40) | 4.01 (3.57, 4.51) | <0.0001* |
| I48.9 | Atrial fibrillation and atrial flutter, unspecified (I48.9) | 761 (0.72) | 368 (0.34) | 2.13 (1.86, 2.45) | <0.0001* |
| I73.9 | Peripheral vascular disease, unspecified (I73.9) | 555 (0.54) | 320 (0.30) | 1.78 (1.53, 2.07) | <0.0001* |
| J44.1 | Chronic obstructive pulmonary disease with acute exacerbation, unspecified (J44.1) | 281 (0.27) | 304 (0.28) | 0.95 (0.81, 1.12) | 0.5601 |
| A41.9 | Sepsis, unspecified (A41.9) | 380 (0.36) | 273 (0.26) | 1.43 (1.21, 1.70) | <0.0001 |
| J22 | Unspecified acute lower respiratory infection (J22) | 520 (0.50) | 272 (0.26) | 1.97 (1.68, 2.31) | <0.0001* |
| I50.9 | Heart failure, unspecified (I50.9) | 357 (0.34) | 270 (0.25) | 1.37 (1.16, 1.59) | <0.0001 |
| I11.0 | Hypertensive heart disease with (congestive) heart failure (I11.0) | 321 (0.30) | 243 (0.22) | 1.37 (1.15, 1.61) | <0.0001 |
| I42.0 | Dilated cardiomyopathy (I42.0) | 102 (0.10) | 231 (0.21) | 0.45 (0.36, 0.57) | <0.0001* |
| I26.9 | Pulmonary embolism without mention of acute cor pulmonale (I26.9) | 348 (0.33) | 198 (0.18) | 1.81 (1.49, 2.20) | <0.0001* |
| K70.9 | Alcoholic liver disease, unspecified (K70.9) | 62 (0.06) | 159 (0.15) | 0.40 (0.30, 0.54) | <0.0001* |
| X59.9 | Exposure to unspecified factor - Unspecified place (X59.9) | 338 (0.33) | 153 (0.15) | 2.27 (1.85, 2.79) | <0.0001* |
| J47 | Bronchiectasis (J47) | 232 (0.22) | 139 (0.13) | 1.72 (1.39, 2.13) | <0.0001* |
| I35.9 | Aortic valve disorder, unspecified (I35.9) | 118 (0.11) | 136 (0.13) | 0.89 (0.70, 1.14) | 0.3754 |
| I38 | Endocarditis, valve unspecified (I38) | 224 (0.21) | 128 (0.12) | 1.81 (1.45, 2.24) | <0.0001* |
| I25.5 | Ischaemic cardiomyopathy (I25.5) | 41 (0.04) | 123 (0.11) | 0.34 (0.24, 0.49) | <0.0001* |
| C80.9 | Malignant neoplasm, primary site unspecified (C80.9) | 90 (0.09) | 108 (0.1) | 0.86 (0.65, 1.14) | 0.2873 |
| I34.0 | Mitral (valve) insufficiency (I34.0) | 123 (0.12) | 98 (0.09) | 1.29 (0.99, 1.69) | 0.0569 |
| I42.9 | Cardiomyopathy, unspecified (I42.9) | 40 (0.04) | 97 (0.09) | 0.42 (0.29, 0.61) | <0.0001* |
| L03.9 | Cellulitis, unspecified (L03.9) | 159 (0.15) | 92 (0.08) | 1.78 (1.38, 2.31) | <0.0001* |
| I51.7 | Cardiomegaly (I51.7) | 67 (0.06) | 87 (0.08) | 0.79 (0.58, 1.09) | 0.1556 |
| I05.9 | Mitral valve disease, unspecified (I05.9) | 168 (0.16) | 66 (0.06) | 2.63 (1.98, 3.49) | <0.0001* |
| E11.5 | Type 2 diabetes mellitus - With peripheral circulatory complications (E11.5) | 56 (0.05) | 65 (0.06) | 0.89 (0.62, 1.27) | 0.5155 |
| I33.0 | Acute and subacute infective endocarditis (I33.0) | 42 (0.04) | 62 (0.06) | 0.70 (0.47, 1.03) | 0.0724 |
| K70.4 | Alcoholic hepatic failure (K70.4) | 11 (0.01) | 61 (0.06) | 0.19 (0.10, 0.35) | <0.0001* |
| E14.2 | Unspecified diabetes mellitus - With renal complications (E14.2) | 51 (0.05) | 58 (0.05) | 0.91 (0.62, 1.32) | 0.6092 |
| I13.2 | Hypertensive heart and renal disease with both (congestive) heart failure and renal failure (I13.2) | 64 (0.06) | 58 (0.05) | 1.14 (0.80, 1.62) | 0.4768 |
| K85.9 | Acute pancreatitis, unspecified (K85.9) | 59 (0.06) | 56 (0.05) | 1.09 (0.75, 1.57) | 0.6575 |
| E11.2 | Type 2 diabetes mellitus - With renal complications (E11.2) | 47 (0.04) | 55 (0.05) | 0.88 (0.60, 1.30) | 0.5236 |
| I24.9 | Acute ischaemic heart disease, unspecified (I24.9) | 61 (0.06) | 55 (0.05) | 1.14 (0.79, 1.65) | 0.4708 |
| E85.4 | Organ-limited amyloidosis (E85.4) | 23 (0.02) | 44 (0.04) | 0.54 (0.33, 0.89) | 0.0163 |
| I51.8 | Other ill-defined heart diseases (I51.8) | 31 (0.03) | 40 (0.04) | 0.80 (0.50, 1.28) | 0.3483 |
| J86.9 | Pyothorax without fistula (J86.9) | 19 (0.02) | 40 (0.04) | 0.49 (0.28, 0.85) | 0.0104 |
| C83.3 | Diffuse large B-cell lymphoma (C83.3) | 32 (0.03) | 38 (0.03) | 0.87 (0.54, 1.39) | 0.5555 |
| K81.9 | Cholecystitis, unspecified (K81.9) | 52 (0.05) | 37 (0.03) | 1.45 (0.98, 2.21) | 0.0846 |
| L03.1 | Cellulitis of other parts of limb (L03.1) | 100 (0.09) | 34 (0.03) | 3.03 (2.06, 4.48) | <0.0001* |
| I27.2 | Other secondary pulmonary hypertension (I27.2) | 62 (0.06) | 32 (0.03) | 2.00 (1.30, 3.06) | 0.0015 |
| E14.7 | Unspecified diabetes mellitus - With multiple complications (E14.7) | 24 (0.02) | 26 (0.02) | 0.95 (0.55, 1.66) | 0.8611 |
| I35.1 | Aortic (valve) insufficiency (I35.1) | 18 (0.02) | 25 (0.02) | 0.74 (0.41, 1.36) | 0.335 |
| I61.4 | Intracerebral haemorrhage in cerebellum (I61.4) | 19 (0.02) | 25 (0.02) | 0.78 (0.43, 1.42) | 0.4228 |
| I42.6 | Alcoholic cardiomyopathy (I42.6) | 1 (0) | 24 (0.02) | 0.04 (0.01, 0.32) | 0.002 |
| E11.7 | Type 2 diabetes mellitus - With multiple complications (E11.7) | 18 (0.02) | 23 (0.02) | 0.81 (0.44, 1.50) | 0.4952 |
| I27.0 | Primary pulmonary hypertension (I27.0) | 28 (0.03) | 23 (0.02) | 1.26 (0.72, 2.18) | 0.4194 |
| G93.1 | Anoxic brain damage, not elsewhere classified (G93.1) | 14 (0.01) | 22 (0.02) | 0.66 (0.34, 1.28) | 0.2176 |
| I77.6 | Arteritis, unspecified (I77.6) | 21 (0.02) | 22 (0.02) | 0.98 (0.54, 1.79) | 0.9582 |
| I08.0 | Disorders of both mitral and aortic valves (I08.0) | 39 (0.04) | 21 (0.02) | 1.92 (1.13, 3.26) | 0.0164 |
| E66.8 | Other obesity (E66.8) | 23 (0.02) | 18 (0.02) | 1.32 (0.71, 2.44) | 0.3811 |
| E66.9 | Obesity, unspecified (E66.9) | 19 (0.02) | 18 (0.02) | 1.09 (0.57, 2.07) | 0.797 |
| I07.1 | Tricuspid insufficiency (I07.1) | 24 (0.02) | 18 (0.02) | 1.37 (0.75, 2.53) | 0.3074 |
| K76.0 | Fatty (change of) liver, not elsewhere classified (K76.0) | 19 (0.02) | 18 (0.02) | 1.09 (0.57, 2.07) | 0.797 |
| C91.0 | Acute lymphoblastic leukaemia [ALL] (C91.0) | 17 (0.02) | 17 (0.02) | 1.03 (0.53, 2.02) | 0.9291 |
| G71.1 | Myotonic disorders (G71.1) | 10 (0.01) | 16 (0.01) | 0.64 (0.29, 1.42) | 0.2756 |
| I27.9 | Pulmonary heart disease, unspecified (I27.9) | 10 (0.01) | 16 (0.01) | 0.64 (0.29, 1.42) | 0.2756 |
| I49.9 | Cardiac arrhythmia, unspecified (I49.9) | 16 (0.02) | 16 (0.01) | 1.03 (0.52, 2.06) | 0.9312 |
| E85.9 | Amyloidosis, unspecified (E85.9) | 10 (0.01) | 15 (0.01) | 0.69 (0.31, 1.53) | 0.3584 |
| I42.2 | Other hypertrophic cardiomyopathy (I42.2) | 13 (0.01) | 15 (0.01) | 0.89 (0.43, 1.88) | 0.7664 |
| J13 | Pneumonia due to Streptococcus pneumoniae (J13) | 13 (0.01) | 15 (0.01) | 0.89 (0.43, 1.88) | 0.7664 |
| N00.9 | Acute nephritic syndrome - Unspecified (N00.9) | 4 (0) | 15 (0.01) | 0.27 (0.09, 0.83) | 0.0217 |
| I34.1 | Mitral (valve) prolapse (I34.1) | 13 (0.01) | 14 (0.01) | 0.96 (0.45, 2.04) | 0.9099 |
| U99 | Codes for research or others (U99) | 8 (0.01) | 14 (0.01) | 0.59 (0.25, 1.40) | 0.2325 |
| I49.0 | Ventricular fibrillation and flutter (I49.0) | 5 (0) | 13 (0.01) | 0.40 (0.14, 1.11) | 0.0788 |
| K63.9 | Disease of intestine, unspecified (K63.9) | 16 (0.02) | 13 (0.01) | 1.27 (0.61, 2.64) | 0.5236 |
| M31.3 | Wegener granulomatosis (M31.3) | 7 (0.01) | 13 (0.01) | 0.56 (0.22, 1.39) | 0.2093 |
| D47.4 | Osteomyelofibrosis (D47.4) | 10 (0.01) | 12 (0.01) | 0.86 (0.37, 1.99) | 0.7229 |
| I31.1 | Chronic constrictive pericarditis (I31.1) | 1 (0) | 12 (0.01) | 0.09 (0.01, 0.66) | 0.0184 |
| I31.9 | Disease of pericardium, unspecified (I31.9) | 4 (0) | 12 (0.01) | 0.34 (0.11, 1.07) | 0.0643 |
| I61.5 | Intracerebral haemorrhage, intraventricular (I61.5) | 18 (0.02) | 12 (0.01) | 1.55 (0.75, 3.21) | 0.242 |
| Q61.3 | Polycystic kidney, unspecified (Q61.3) | 11 (0.01) | 12 (0.01) | 0.95 (0.42, 2.14) | 0.8923 |
| Y83.5 | Amputation of limb(s) (Y83.5) | 7 (0.01) | 12 (0.01) | 0.60 (0.24, 1.53) | 0.285 |
| K22.5 | Diverticulum of oesophagus, acquired (K22.5) | 8 (0.01) | 11 (0.01) | 0.75 (0.30, 1.86) | 0.5354 |
| C93.1 | Chronic myelomonocytic leukaemia (C93.1) | 10 (0.01) | 10 (0.01) | 1.03 (0.43, 2.48) | 0.9456 |
| I42.1 | Obstructive hypertrophic cardiomyopathy (I42.1) | 8 (0.01) | 10 (0.01) | 0.82 (0.33, 2.09) | 0.6847 |
| K66.0 | Peritoneal adhesions (K66.0) | 19 (0.02) | 10 (0.01) | 1.96 (0.91, 4.21) | 0.0852 |
| E05.9 | Thyrotoxicosis, unspecified (E05.9) | 24 (0.02) | 9 (0.01) | 2.75 (1.28, 5.92) | 0.0097 |
| I05.0 | Mitral stenosis (I05.0) | 47 (0.04) | 9 (0.01) | 5.39 (2.64, 10.99) | <0.0001* |
| I42.5 | Other restrictive cardiomyopathy (I42.5) | 6 (0.01) | 9 (0.01) | 0.69 (0.24, 1.93) | 0.4768 |
| Y83.1 | Surgical operation with implant of artificial internal device (Y83.1) | 13 (0.01) | 9 (0.01) | 1.49 (0.64, 3.48) | 0.3584 |
| C50.9 | Malignant neoplasm: Breast, unspecified (C50.9) | 1201 (1.16) | 8 (0.01) | 149.44 (71.15, 313.90) | <0.0001* |
| G71.0 | Muscular dystrophy (G71.0) | 6 (0.01) | 8 (0.01) | 0.77 (0.27, 2.23) | 0.6339 |
| K70.1 | Alcoholic hepatitis (K70.1) | 6 (0.01) | 8 (0.01) | 0.77 (0.27, 2.23) | 0.6339 |
| K80.3 | Calculus of bile duct with cholangitis (K80.3) | 8 (0.01) | 8 (0.01) | 1.03 (0.39, 2.75) | 0.9513 |
| W06.0 | Fall involving bed - Home (W06.0) | 5 (0) | 8 (0.01) | 0.64 (0.21, 1.97) | 0.4407 |
| Y83.8 | Other surgical procedures (Y83.8) | 13 (0.01) | 8 (0.01) | 1.68 (0.69, 4.04) | 0.2508 |
| A41.2 | Sepsis due to unspecified staphylococcus (A41.2) | 11 (0.01) | 7 (0.01) | 1.62 (0.63, 4.18) | 0.3183 |
| B90.9 | Sequelae of respiratory and unspecified tuberculosis (B90.9) | 6 (0.01) | 7 (0.01) | 0.88 (0.30, 2.63) | 0.8241 |

Note: P-values satisfying Bonferroni correction threshold 0.05/2432 = 2.039 × 10^-5^
